# An approach to forecast human cancer by profiling microRNA expressions from NGS data

**DOI:** 10.1186/s12885-016-3042-2

**Published:** 2017-01-25

**Authors:** A. Salim, R. Amjesh, S. S. Vinod Chandra

**Affiliations:** 1 Department of Computer Science, College of Engineering Trivandrum, Sreekaryam, Thiruvananthapuram, India; 2 Department of Computational Biology and BioInformatics, University of Kerala, Karyavattom, Thiruvananthapuram, India; 3 Computer Center, University of Kerala, Thiruvananthapuram, India

**Keywords:** MicroRNA, Expression profiling, Sequence mapping, SVM classifiers

## Abstract

**Background:**

microRNAs are single-stranded non-coding RNA sequences of 18 - 24 nucleotides in length. They play an important role in post-transcriptional regulation of gene expression. Evidences of microRNA acting as promoter/suppressor of several diseases including cancer are being unveiled. Recent studies have shown that microRNAs are differentially expressed in disease states when compared with that of normal states. Profiling of microRNA is a good measure to estimate the differences in expression levels, which can be further utilized to understand the progression of any associated disease.

**Methods:**

Machine learning techniques, when applied to microRNA expression values obtained from NGS data, could be utilized for the development of effective disease prediction system. This paper discusses an approach for microRNA expression profiling, its normalization and a Support Vector based machine learning technique to develop a Cancer Prediction System. Presently, the system has been trained with data samples of hepatocellular carcinoma, carcinomas of the bladder and lung cancer. microRNAs related to specific types of cancer were used to build the classifier.

**Results:**

When the system is trained and tested with 10 fold cross validation, the prediction accuracy obtained is 97.56% for lung cancer, 97.82% for hepatocellular carcinoma and 95.0% for carcinomas of the bladder. The system is further validated with separate test sets, which show accuracies higher than 90%. A ranking based on differential expression marks the relative significance of each microRNA in the prediction process.

**Conclusions:**

Results from experiments proved that microRNA expression profiling is an effective mechanism for disease identification, provided sufficiently large database is available.

**Electronic supplementary material:**

The online version of this article (doi:10.1186/s12885-016-3042-2) contains supplementary material, which is available to authorized users.

## Background

microRNAs belong to the family of non-coding RNAs, having length around 22 nucleotides and are found in many eukaryotes including human beings [1]. Recent studies have identified evidences of its role in wide variety of biological processes such as normal cell development, differentiation, growth control and progression/suppression of many diseases including cancer [2]. Mature microRNAs may make Watson-Crick base pairing to the 3’ untranslated region of mRNA, causing gene expression regulation [3–5]. In fact, the gene regulation is by mRNA degradation or by repression of mRNA translation process [6–8]. Studies have undeniably proved the difference in expression levels of microRNAs in normal and diseased conditions [9, 10]. Thus, the role of microRNAs in gene regulation is in turn associated with the metamorphosis of diseases. Study of microRNAs and its connection with various diseases lead to developments in targeted therapy against specific molecular activity [11]. Recent studies have revealed that several microRNAs are acting similar to oncogenes / tumour suppressors. Initially identified tumour suppressor microRNAs include miR 143, miR-145, miR-15a, miR-16-1 and let-7 family members, whereas oncogenes include miR-21, miR-221 and miR-155 [12]. Over expression of hsa-Mir-101 inhibits spreading of lung cancer by reduction in the gene activity of zeste homolog2 (EZH2) [13], whereas reduced expression of hsa-let-7c is associated with shorter survival of lung cancer patients [14]. Expression of *β*−*c* *a* *t* *e* *n* *i* *n* can be regulated by microRNA-33a which results in lung cancer cell proliferation [15]. Downregulation of lung cancer by hsa-mir-30c due to its targeted activity against Rab 18 gene was proved by a qRT-PCR profiling experiment [16]. Landi et al. reported a scheme with signature of five microRNAs (miR-25, miR-34c-5p, miR-191, let-7e and miR-34a) to differentiate both the sub types of lung cancer, Adenocarcinoma (AD) and Squamous cell carcinoma (SCC) [17]. Aberrant expression of several microRNAs were correlated with bio-pathological and clinical features of hepatocellular carcinoma (HCC). Over expression of microRNAs were linked to cancer-associated pathways, indicating a direct role in liver tumorigenesis. For example, upregulation of miR-221 and miR-21 promotes cell cycle progression, reduces cell death and favours angiogenesis and invasion. These findings suggest that microRNAs can be novel molecular targets for HCC treatment [18]. Presently, efforts have been made by researchers to collect and publish association between microRNAs and various diseases by text mining the available literature. MiRCancer is one such database extracted from literature, shows 878 associations between 79 human cancers and 236 microRNAs [19]. PhenomiR is yet another manually curated database, where deregulation of microRNAs in diseases is investigated from 542 studies [20].

microRNA profiling application ranges from identification of microRNAs involved in cell differentiation, novel microRNA discovery, microRNA : mRNA and microRNA : protein interactions and as biomarkers. It is a difficult task due to very low presence (0.01%) of microRNA in total RNA mass, lack of common start or stop sequence and very short sequence length. Despite these challenges, three different strategies were established - a hybridization based method (microarray, nCounter), Quantitative reverse transcription PCR (qRT-PCR) and Next Generation Sequencing (RNA-Seq) [21]. qRT-PCR is suitable for absolute quantification, but less capable to identify novel microRNAs. Microarray is a high throughput operation with low cost, but absolute quantification is difficult. RNA-Seq ensures high accuracy in distinguishing microRNA with similar sequences and thereby novel microRNAs can also be detected. Shirley et al. compared different profiling systems and concluded that NGS platforms have highest detection sensitivity, highest differential expression analysis accuracy and high level of technical re-productivity [22]. Data analysis of Next Generation Sequencing (NGS) consists of several steps. A generalized NGS pipeline begins with a preprocessing, where adapter contamination is removed and low quality reads are trimmed. Next step is mapping of reads to a reference sequence. Depending upon application, the reference sequence can be either a genome or a transcript.

NGS reads may contain adapter or fragments of adapter sequence, which were added during library preparation step of sequence generation. Adapters are not part of biological sequence and if not trimmed would be a reason for wrong downstream analysis. Given a read and an adapter sequence the problem of adapter removal can be modelled as an optimal semi global sequence alignment problem. Several of preprocessing tools have been developed recently for efficient adapter removal and quality trimming. They differ in accuracy, speed, memory requirement, capability to trim at 5’ end or 3’ end or both the ends and capable of handling single or paired end reads. Quite a few algorithms are based on Watermann Smith sequence alignment algorithm, having a time complexity of *O*(*m* *n*). Btrim [23] is a very fast tool that works with a time complexity of *O*(*m* *n*/*w*), where *w* is word length of the computer. FastX, TagCleaner [24] and SeqTrim [25] are useful only for single end reads. Besides handling of both single and paired end reads, low quality trimming can be performed with Trimomatic [26], AllenTrimmer [27], Cutadapt [28], AdapterRemoval [29] and Skewer [30].

In genome scale mapping, the reference sequence consists of millions of nucleotides, there could be many locations where a *read* has an approximate/exact match. Around 60 sequence mapping tools were developed after Next Generation Sequencing came into existence. Accuracy, speed, read length support and memory requirement are critical parameters in measuring performance of sequence mapping tools. Speed of operation can be enhanced by applying limits to the number of mismatches permitted and the gap lengths allowed. Another possible way to increase speed is by ignoring read quality score and single nucleotide polymorphism(SNP) information. The number of mapped reads decreases with increase in read length for a given threshold of mismatches permitted. Paired ends map to a reference sequence if the reads are within a threshold value of insert size. Throughput of tools that consider paired ends are lesser than those of single end reads [31].

Generally, sequence mapping starts with the creation of an index of the reference sequence. Most popular indexing techniques used in the tools are hash table and Burrows Wheeler Transform (BWT). Hash table based indexing keeps a pair - keyword and value, where keyword is a k-mer generated from the reference sequence and value returned is the coordinate of matched location in the reference sequence. A new index with small memory requirement, based on BWT, namely FM Index is the backbone of another set of tools. When compared with Hash tables, index built time is higher for BWT, but it works efficiently in cases when a single read matches with multiple locations. Examples of tools based on BWT indexing are BWA [32], Bowtie [33] and SOAP2 [34], whereas SOAP [35], NovoAlign [36], and mrsFast [37] are tools based on Hash table. Majority of the algorithms consider first few tens of base pairs of a read as seed region. It is relevant due to the fact that chances of errors in base pairing is feeble in this region. The number of mismatches allowed in seed region, length of seed region, number of mismatches allowed in non seed region are the main input parameters in mapping tools.

The objective of the present study is to develop a cancer prediction system using microRNA profiling. This is accomplished by the application of machine learning technique on differential expression of specific set of microRNAs in normal and in tumour samples. Profiling of microRNAs is performed by removing the adapter sequences, sequence mapping to quantify mature microRNA sequences, followed by a normalization procedure.

## Methods

### Data collection

microRNA transcriptome data for lung cancer, hepatocellular carcinoma and bladder cancer were used to build a cancer prediction system. Data in Sequence Read Archives (SRA)format was downloaded from National Center for Biotechnology Information (NCBI). The lung cancer data set consists of 41 samples (SRP009408- microRNA expression profiles in lung cancer tissues versus adjacent lung tissues using next-gen sequencing). 20 samples of bladder cancer (SRP007946) and 46 samples of both normal and tumour for hepatocellular carcinoma (SRP049590) were downloaded. Illumina Genome Analyzer II was the sequencing machine in all these three cases. Another 9 samples of lung cancer were downloaded to conduct an independent test (SRP040720). The microRNAs that are linked with up/downregulation of lung cancer, hepatocellular carcinoma and carcinomas of the bladder were obtained from microRNA - disease association databases such as miRCancer [19], PhenomiR [20] and from review articles on specific types of cancer [38]. Mature microRNA sequences were downloaded from miRBase [39].

### MicroRNA profiling

Quantification of mature microRNAs is preferred to pre-microRNAs since the former show active role in gene regulation. Several approaches have been employed by researchers/scientists to quantify microRNAs in NGS reads. In one of the profiling experiments, mature microRNA sequence aligned to a *read* with a maximum of one mismatch was considered as a *hit* [40]. Two or three mismatches for longer reads were allowed in other experiments [41]. There are examples of studies with a restriction that exact match between a *read* and a mature microRNA were made mandatory to prevent the reads mapped to paralogs of a given microRNA and to avoid multiple ambiguous hits [42].

**Fig. 1:**
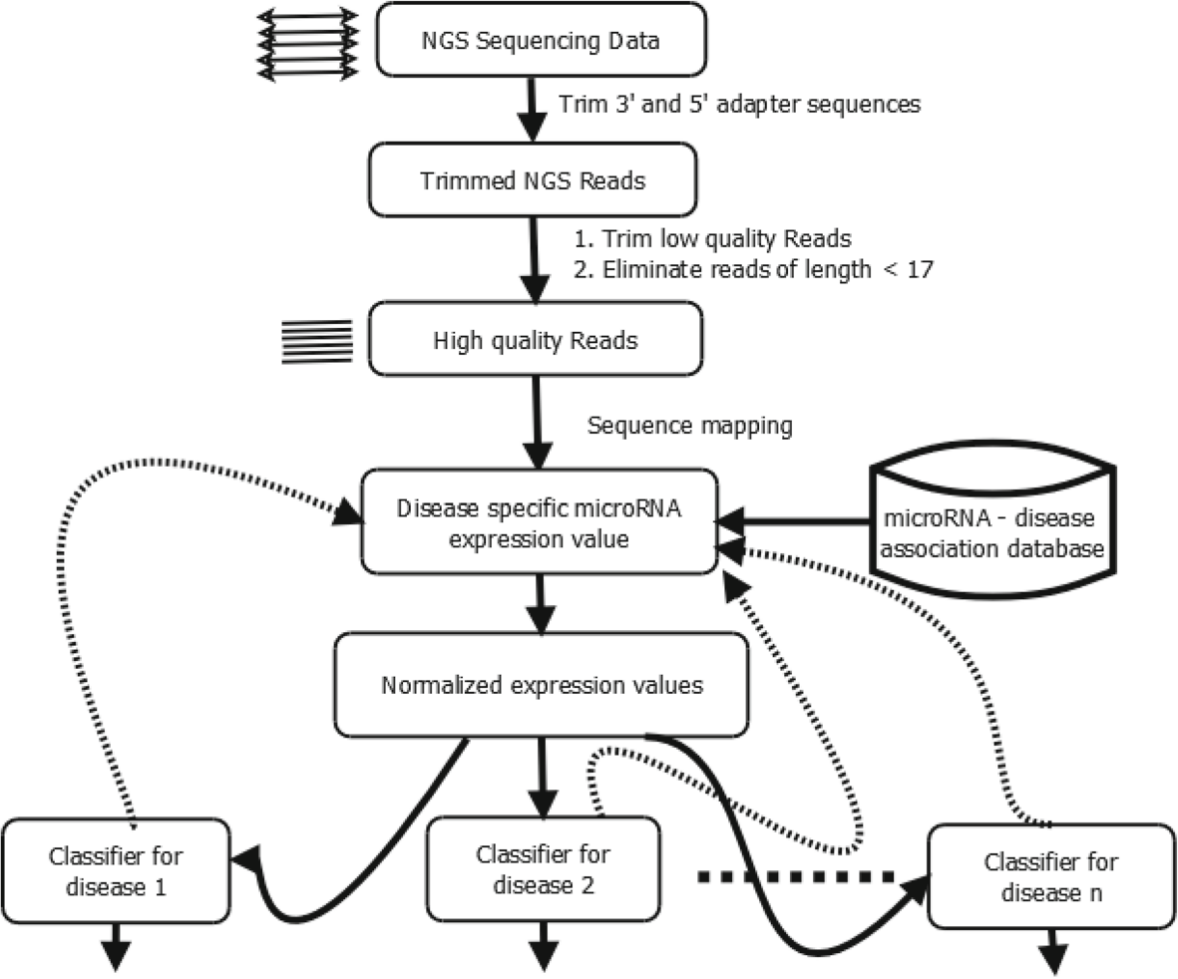
General architecture of cancer prediction system

The proposed architecture for cancer prediction system from NGS data is depicted in Fig. 1. The sequence of operations involved in the process is; 1) reads are preprocessed so that they become devoid of adapter contamination and satisfy minimum quality threshold and length. 2) Resultant reads are aligned to MirBase V 20. 3) Quantification of disease specific microRNAs from the samples is determined. 4) Normalize the read counts 5) Apply machine learning technique to build a classifier.

To perform preprocessing we used *TrimGalore*, which in effect uses two popular tools *cutadapt* and *FastQC*. Adapter removal was done by *cutadapt* and quality trimming was done by *FastQC*. In this experiment, we insist the length of resultant reads to be atleast 20 and the quality threshold to be at least 30. To align reads to MirBase, a memory efficient and ultra fast sequence mapping tool, Bowtie is used [33]. Memory efficiency and speed are attained by creating an index of the reference sequence. Bowtie equipped with a tool, *b* *o* *w* *t* *i* *e*−*b* *u* *i* *l* *d*, to create the index. Bowtie alignment policy can be set either by seed length (-l) and number of mismatches in seed region (-n) or total number of mismatches (-v) in the entire alignment. When a sequence mapping tool like Bowtie is used to map reads to a mature microRNA sequence, the total number of aligned *reads* can be taken as the measure of its expression level. We used *idxstats* of samtools [43] to get the statistics of mapped and unmapped reads against each microRNA.

### Expression normalization

In microRNA profiling, normalization is a critical step as it tries to correct bias in the data. Several normalization methods are available, specifically applicable to microarray analysis, Real time PCR and Next Generation Sequencing. In Next Generation Sequencing, relative count of microRNA is found by normalizing reads against total number of reads in the sample or total number of maps to microRNAs in the sample. The resultant value is expressed as reads per million to respective library. Z-score normalization determines the variation of expression value from the mean in units of standard deviation. In this experiment, the normalized expression of a microRNA is Z-score value of microRNA expression with respect to the total mapped microRNAs in the sample.

### Differential expression of microRNAs

Normalized expression values of a microRNA from all samples can be viewed as a vector of *n* dimensions, where *n* is the number of samples. Differential expression in normal and tumour sample was obtained by finding Euclidean distance as a measure of degree of difference in expression values between the samples. If *P* = (*x*_1_,*x*_2_,……,*x*_*n*_) and *Q* = (*y*_1_,*y*_2_,……,*y*_*n*_) are two vectors, the distance between P and Q is obtained by $d = \sqrt {\Sigma _{i=1}^{n} \left (x_{i}-y_{i}\right)^{2}}$.

### Prediction model by SVM

A prediction model based on Support Vector Machine(SVM) is used to classify the data samples. SVM is a supervised machine learning algorithm. It works by projecting data in input space to a feature space of higher dimensions. SVM has been selected for this experiment due to its ability to handle high dimensional data and due to its higher prediction accuracy. A linear classifier is based on a discriminant function of the form *f*(*x*) = *ω* ^*T*^ *x* + *b*, where *ω* is weight vector and *b* is bias. *ω* ^*T*^ *x* is dot product between two vectors and it is defined as *ω* ^*T*^ *x*=*Σ*_*i*_ *ω*_*i*_ *x*_*i*_. A hyperplane is the set of all points with *ω* ^*T*^ *x*= 0, which separates input data into two classes. The bias, *b* translate the hyperplane away from the origin. The closest points to the hyperplane among positive and negative samples define a *margin*. Instances in the training set can be viewed as pair (*x*_*i*_, *y*_*i*_) ∀*i*=1, *m*, where *m* is number of instances in the training set. SVM minimizes the risk of misclassification by maximizing the margin between the data points. Therefore, SVM is basically an optimization problem to find out a Lagrangian multiplier, *α*_*i*_>0, such that *L* is maximum with a constraint.

$$\begin{aligned} &\text{maximize} & L~=~\sum_{i=1}^{m} \alpha_{i} -\frac{1}{2}\sum_{i,j=1}^{m}\alpha_{i}\alpha_{j}y_{i}y_{j}(x_{j}.x_{i}) \\ & \text{subject to} & \sum_{i=1}^{m} \alpha_{i}y_{i}=0,~~~~ \alpha_{i} \geq 0,~~i~=~1,\ldots,m \end{aligned} $$

A linear SVM is of no use, if input data instances are not separable by a linear boundary. Solution to this problem is mapping of data in to a higher dimensional space which may exhibit a linear pattern. A non-linear SVM classifier is based on discriminant function of form *f*(*x*)=*ω* ^*T*^ *ϕ*(*x*) + *b*, where *ϕ* is a non-linear function. Direct computation of the function *ϕ* is not scalable with the number of input features. An efficient way of computation known as kernel trick *k* is employed to limit the size of resultant feature space and thus memory and computational requirements. A Pearson VII kernel(PUK) is defined as

$$K(x, y) =\frac{1}{{\left(1+{\left(\frac{2~\sqrt{{\|x~-~y\|}^{2}~\sqrt{2^{\left(\frac{1}{\omega}\right)}-1}}}{\sigma}\right)}^{2}\right)}^{\omega}} $$

where *ω* and *σ* control half width and trailing factor of peak, respectively.

We trained and tested classifiers for lung cancer, carcinomas of the bladder and hepatocellular carcinoma separately. We evaluated the performance of the models with three different kernel functions, namely Normalized polynomial kernel, RBF Kernel and Pearson VII kernel(PUK). Normalized polynomial kernel and Pearson VII kernel(PUK) functions were giving almost same performance. The performance of the classifier model was evaluated with the following measures:

$$\begin{aligned} precision = \frac{TP}{TP+FP} \\ True~ Positive~ Rate /Recall / Sensitivity = \frac{TP}{TP+FN}\\ False~ Positive~ Rate = \frac{FP}{TN+FP}\\ Accuracy = \frac{TP+TN}{TP+TN+FP+FN} \\ \end{aligned} $$

## Results

microRNA data with respect to sequencing experiments in lung cancer, hepatocellular carcinoma and carcinomas of the bladder were retrieved from NCBI. The lung cancer data set contains 21 positive and 20 negative samples, whereas the data sets of hepatocellular carcinoma contains 23 positive and 23 negative samples, and the data sets of carcinomas of the bladder contains 10 positive and 10 negative samples. Accession codes and total number of reads in each sample are given in Additional file 1. microRNAs associated to each type of cancer were obtained from the disease association databases such as miRCancer and Phinomir as well as collected manually from the literature. List of microRNAs used in our study is given in Additional file 2. NGS data were pre-processed to remove adapter sequences as well as to satisfy strict read length and base pair quality threshold. The pre-processed samples with number of quality reads are given in Additional file 3. Cancer-specific microRNAs were mapped against quality reads using Bowtie. Expression values obtained were normalized using Z-Score normalization. Additional file 4 contains normalized expression values of the respective microRNAs associated with lung cancer. Similarly, Additional files 5 and 6 show the same information associated with hepatocellular carcinoma and carcinomas of the bladder.

Differential expression of a microRNA was computed as the Euclidean distance between expression values in normal and tumour samples. microRNAs were ranked by arranging them in descending order of differential expression. Table 1 shows the list of top ranked 20 microRNAs in each type of cancer. These results correlate with proved role of microRNAs in different experiments. For instance, over expression of miR 122 downregulates hepatocellular carcinoma by controlling the expression of Wnt 1, *β*-catenin and TCF-4 [44], which is ranked 1^*st*^ in our experiment. The second ranked microRNA is hsa-miR-21. Clinical evidence shows that miR-21 acts as tumour suppressor by targeting MAP2K3 gene [45]. microRNA profiles for lung cancer diagnosis and prognosis have been studied by Yanaihara et al. [46] and listed 43 differentially expressed microRNAs. hsa-miR-21 is one among them, and is listed top in the lung cancer samples of our study. hsa-miR-143 has potential role in predicting survival of bladder cancer patients [47]. Our study shows that hsa-miR-143 is the most widely differed microRNA in bladder cancer data set.

**Table 1:** microRNAs associated with lung cancer, hepatocellular carcinoma and carcinomas of the bladder

Rank	Lung cancer	Hepatocellular carcinoma	Carcinomas of the bladder
1	hsa-miR-21-5p	hsa-miR-122-5p	hsa-miR-143-3p
2	hsa-miR-148a-3p	hsa-miR-21-5p	hsa-miR-200c-3p
3	hsa-let-7g-5p	hsa-miR-143-3p	hsa-miR-182-5p
4	hsa-miR-101-3p	hsa-miR-148a-3p	hsa-miR-146b-5p
5	hsa-miR-103a-3p	hsa-miR-101-3p	hsa-miR-103a-3p
6	hsa-miR-29a-3p	hsa-miR-199b-3p	hsa-miR-183-5p
7	hsa-miR-23a-3p	hsa-let-7g-5p	hsa-miR-200b-3p
8	hsa-let-7i-5p	hsa-miR-30d-5p	hsa-miR-29c-3p
9	hsa-miR-199b-3p	hsa-miR-100-5p	hsa-miR-145-5p
10	hsa-miR-146a-5p	hsa-let-7i-5p	hsa-miR-205-5p
11	hsa-miR-186-5p	hsa-miR-125b-5p	hsa-miR-200a-3p
12	hsa-miR-200a-3p	hsa-miR-30a-5p	hsa-miR-141-3p
13	hsa-let-7d-5p	hsa-miR-145-5p	hsa-miR-126-3p
14	hsa-let-7c-5p	hsa-miR-29a-3p	hsa-miR-99a-5p
15	hsa-miR-135b-5p	hsa-miR-182-5p	hsa-miR-16-5p
16	hsa-let-7e-5p	hsa-miR-200a-3p	hsa-miR-26a-5p
17	hsa-miR-17-5p	hsa-miR-23a-3p	hsa-miR-23a-3p
18	hsa-miR-19b-3p	hsa-let-7c-5p	hsa-miR-26b-5p
19	hsa-miR-1-3p	hsa-miR-146a-5p	hsa-miR-10b-5p
20	hsa-miR-194-5p	hsa-miR-125a-5p	hsa-miR-185-5p

List is in the decreasing order of euclidean distance values between expression levels of normal and tumour samples

**Fig. 2:**
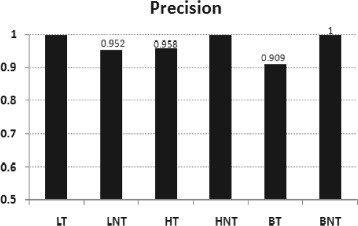
Performance measures of cancer prediction systems: precision - obtained by SVMs with Pearson VII kernel(PUK) (*c*=1, *ε*_*i*_=1×10^−12^,*ω*=1, *σ*=1), when 10 fold cross validation test is performed on input data. LT and LNT: Positive and negative samples of lung cancer, BT and BNT: Positive and negative samples of carcinomas of the bladder, HT and HNT : Positive and negative hepatocellular carcinoma

**Fig. 3:**
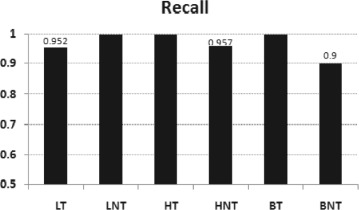
Performance measures of cancer prediction systems: recall - obtained by SVMs with Pearson VII kernel(PUK) (*c*=1, *ε*_*i*_=1×10^−12^,*ω*=1, *σ*=1), when 10 fold cross validation test is performed on input data. LT and LNT: Positive and negative samples of lung cancer, BT and BNT: Positive and negative samples of carcinomas of the bladder, HT and HNT : Positive and negative hepatocellular carcinoma

The cancer prediction system were developed using Support Vector based classifier model. When the system is trained and tested with 10 fold cross validation, the prediction accuracy is 97.56% for lung cancer, 97.82% for hepatocellular carcinoma and 95.0% for carcinomas of the bladder. Figure 2 shows the *precision* and Fig. 3 shows the *recall* obtained from the experiment. Predicted results did not contain any false positive in the case of positive samples of lung cancer, negative samples of hepatocellular carcinoma and carcinomas of the bladder.

Even though cross validation is an effective method for validation when limited number of samples are available, we verified the developed model using separate test set and independent test set. Out of 41 samples of lung cancer, 32 were used for training the model and the remaining 9 samples were used to test it. There was only one wrong prediction, which was a false negative prediction. Similarly, for hepatocellular carcinoma we trained with 33 instances and tested the model using 13 instances. Again there was only one wrong prediction and the accuracy is 92.8%. Table 2 shows the values obtained for different performance measures on separate test sets. Further, we have used 9 lung cancer samples from another experiment to conduct an independent test (SRP040720). When tested with the model trained by the original 41 samples, all other predictions were correct except two. Similarly for hepatocellular carcinoma, we used another data set containing just 4 samples and there were no wrong predictions (SRP065616). Thus, our model is giving promising result with separate and independent tests. The test samples used for validation are given as Additional file 7. Also, library preparation strategy, source, layout and selection method were the same for the test and the training data.

**Table 2:** Prediction performances when separate training and test data were used

Cancer type	Number of training samples	Number of test samples	TP rate	FP rate	Precision	Recall	Accuracy
Lung cancer	32	9	0.889	0.089	0.911	0.889	0.9
Hepato cellular carcinoma	33	13	0.9375	0.063	0.917	0.938	0.923

**Fig. 4:**
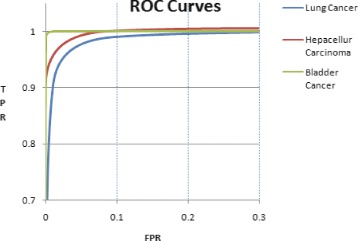
ROC curves: Comparison of ROC curves of three cancer predictors. The highest coverage is for carcinomas of the bladder predictor

We extended the validation of classifier model by increasing the number of negative samples in the data set. microRNA profiling has been repeated for each set of microRNAs specific to each type of cancer. For example, lung cancer specific microRNAs are profiled using data samples of hepatocellular carcinoma and carcinomas of the bladder. Expression values were normalized, appended to the negative set and the cancer prediction system was trained again. Table 3 shows the result obtained from the extended sample set. The resultant accuracy were 97.56%, 97.82% and 100%, respectively for lung cancer, hepatocellular carcinoma and carcinomas of the bladder. Though the number of samples were increased by three fold, precision values obtained were 97.5%, 98.3% and 99.2% and the recall values were 97.5%, 98.3% and 99.2%. Figure 4 shows ROC curves of the experiment conducted with higher number of negative samples. The area under ROC curve is a measure of the discriminatory power of the classifier. In the case of lung cancer samples, True Positive Rate (TPR) touched 0.90 while False Positive Rate (FPR) was just 0.01, and when FPR was less than 0.09, the TPR crossed 0.99.

**Table 3:** Accuracy, precision and recall values obtained when additional negative data samples were used

Cancer types	TP	TN	FP+FN	Accuracy	Precision	Recall
Lung cancer	21	100	3	97.58	0.951	0.967
Hepatocellular carcinoma	24	97	2	98.37	0.975	0.975
Bladder cancer	10	103	1	99.12	0.954	0.991

**Fig. 5:**
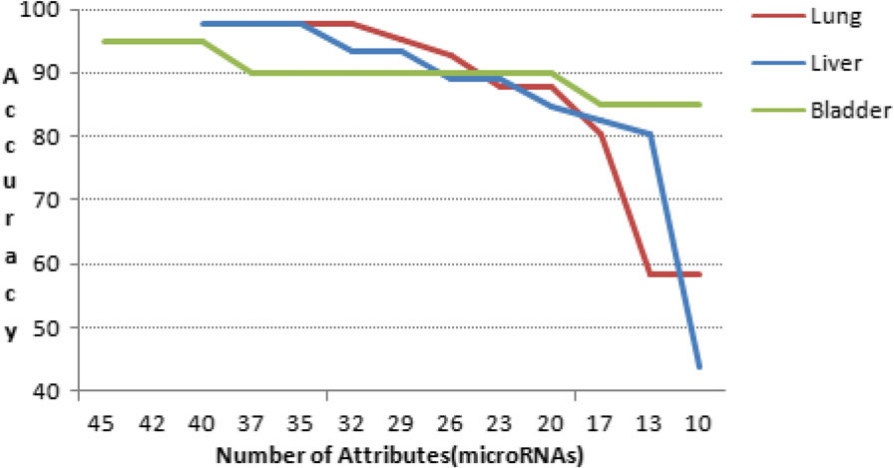
Prediction accuracy versus numbers of attributes (microRNAs). Accuracy of prediction is above 90% when atleast 24 microRNAs are used in the experiment for lung cancer samples, 26 microRNAs for hepatocellular carcinoma samples and 20 microRNAs for carcinomas of the bladder samples

**Fig. 6:**
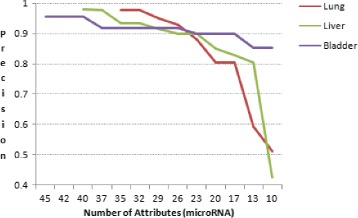
Precision versus number of attributes (microRNAs). Precision of prediction is above 90% when atleast 24 microRNAs are used in the experiment for lung cancer samples, 27 microRNAs for hepatocellular carcinoma samples and 17 microRNAs for carcinomas of the bladder samples

Figures 5 and 6 shows the accuracy and the precision of prediction when number of attributes(microRNA) used in the samples were reduced. We have obtained an accuracy value of 90% or higher, when a minimum of 24, 26 and 17 attributes were used in lung cancer, hepatocellular carcinoma and carcinomas of the bladder samples, respectively. Similarly, to predict with a precision value as 90% or higher, the number of attributes used were 24, 27 and 20 for lung cancer, hepatocellular carcinoma and carcinomas of the bladder samples, respectively.

## Discussion

Early detection is important in the successful treatment of cancer or any other chronic disease. There are many molecular biological techniques available, but they may often expensive and may undergo long diagnostic procedures. We developed a computational method to predict incidences of cancer by using differential expression of specific set of microRNAs. NGS sequencing techniques have evolved to an extent where the cost of experiment is becoming cheaper. This makes NGS based microRNA profiling a feasible option to find aberrant expression of microRNAs. Our prediction system is designed in such a way to predict any type of cancer, provided the system has been trained with data for that particular type of cancer. Presently, the system is capable of predicting three different types of cancer.

One of the challenges in accurate detection and quantification of microRNAs when compared with mRNA profiling is handling of shorter length of mature microRNA sequence. This makes the annealing of primers in reverse transcription and PCR to a difficult process. Another barrier in annealing is the inability to selective enrichment due to the absence of a common sequence and wide variation in melting temperature due to the variance in GC content of microRNAs. An implicit assumption in mRNA profiling studies is that there exists a correlation of protein level and differential expression of mRNAs. Normally, a correlation coefficient of mRNA expression versus protein expression is calculated in genome wide studies [48]. When microRNA profiling data needs to be analysed, same yardsticks such as distribution assumptions developed for a typical mRNA assay are not suitable. The variation in total microRNA levels in different samples and dynamic range of expression levels are challenges to be addressed in microRNA profiling. A single microRNA may interact with several mRNAs and a single mRNA may get affected by several microRNAs. Several computational algorithms have been developed for finding potential target sites. A combined effort of computational and experimental validation of target sites which further extend to the identification of variation in protein level expression might contribute to get a comprehensive insight in tumorigenesis.

Biomarkers help to assess disease status and act as an aid for early diagnosis for many types of cancer [49, 50]. Better results were obtained when combination of multiple biomarkers were used, rather than their individual predictions [51]. Studies related to the use of microRNA as a potential biomarker in cancer diagnosis and prognosis reckoned microRNA profiling as a signature identification scheme [52]. Difference in microRNA expression can be detected from affected tissues, from circulating tumour cells in blood samples and by the detection of exosomic microRNAs in the microenvironment of tumour [53]. To translate the method suggested in this paper into a good clinical alternative for cancer detection, it is essential to fix RNASeq experiment and its parameters for microRNA profiling for specific cancer types. The advantage of algorithm discussed in this paper is that, expression profiling needs to be conducted for a limited number of microRNAs. At the same time, selection of microRNAs associated with each specific cancer type is a very difficult task, as some microRNAs are associated with several diseases.

## Conclusions

The exact molecular mechanism behind gene expression regulation of microRNAs is not unveiled completely. But, increasing evidences with experimental proofs are available for the association between microRNAs and different diseases. The progress in Next Generation Sequencing added great momentum in microRNA research. Many studies related to differential expression of microRNAs in specific diseases/cancer are in literature, but development of cancer prediction system using microRNA profiling is a novel approach.In this paper, we present a method to predict the incidence of cancer by analyzing the NGS data based on disease specific microRNAs. When the experiments were conducted with lung cancer, hepatocellular carcinoma and carcinomas of the bladder samples, the obtained accuracies of prediction were around 97% in cross validation. Independent and separate tests too gave promising results. Thus, profiling of microRNA in any accepted manner is a useful method in forecasting human cancers as well as other diseases in which the system is trained. We hope this could be further extended for the development of more comprehensive prediction systems.

## Additional files

Additional file 1 List of tumour and normal data samples used in the study. (PDF 41 kb)

Additional file 2 List of disease specific microRNAs used in the study. (PDF 30 kb)

Additional file 3 Pre-processed NGS data samples with respect to normal and tumour tissues used in the study. (PDF 43 kb)

Additional file 4 Normalized expression values of specific set of microRNAs associated with the lung cancer. (PDF 75 kb)

Additional file 5 Normalized expression values of specific set of microRNAs obtained with the hepatocellular carcinoma. (PDF 93 kb)

Additional file 6 Normalized expression values of specific set of microRNAs associated with the carcinomas of the bladder. (PDF 43 kb)

Additional file 7 Normalized expression values of microRNAs associated with lung cancer and hepatocellular carcinoma (Test data sets). (PDF 48 kb)

## Abbreviations

HCC: Hepatocellular carcinoma
NGS: Next generation sequencing
SRA: Sequence read archive
SVM: Support vector machines
