# Supplementary material for: An approach to forecast human cancer by profiling microRNA expressions from NGS data
Source: BMC Cancer. 2017 Jan 25;17:77. doi: 10.1186/s12885-016-3042-2 (PMC5267436; doi:10.1186/s12885-016-3042-2)
Supplement: Additional file 1 — List of tumour and normal data samples used in the study. (PDF 41 kb) [file 12885_2016_3042_MOESM1_ESM.pdf]

**Additional File 1: List of Tumour and Normal tissue data samples with respect to Lung Cancer, Hepatocellular Carcinoma and Carcinomas of the bladder**

| **Lung Cancer** | | | | | |
|---|---|---|---|---|---|
| Normal Samples | | | Tumour Samples | | |
| **Sl No** | **SRR list** | **Total Number of Reads** | **Sl No** | **SRR list** | **Total Number of Reads** |
| 1 | SRR372672 | 53,022,826 | 1 | SRR372671 | 41,195,137 |
| 2 | SRR372670 | 48,415,574 | 2 | SRR372669 | 34,754,806 |
| 3 | SRR372668 | 38,287,217 | 3 | SRR372667 | 37,776,488 |
| 4 | SRR372666 | 35,143,921 | 4 | SRR372665 | 37,507,931 |
| 5 | SRR372664 | 33,656,348 | 5 | SRR372663 | 33,544,033 |
| 6 | SRR372662 | 32,770,003 | 6 | SRR372661 | 37,053,587 |
| 7 | SRR372660 | 37,049,180 | 7 | SRR372659 | 37,140,785 |
| 8 | SRR372658 | 33,354,253 | 8 | SRR372657 | 39,753,719 |
| 9 | SRR372656 | 36,446,549 | 9 | SRR372655 | 33,783,427 |
| 10 | SRR372654 | 33,777,251 | 10 | SRR372653 | 32,579,770 |
| 11 | SRR372652 | 25,425,497 | 11 | SRR372651 | 37,486,200 |
| 12 | SRR372650 | 25,709,897 | 12 | SRR372649 | 36,416,257 |
| 13 | SRR372648 | 35,058,620 | 13 | SRR372647 | 36,647,675 |
| 14 | SRR372646 | 35,759,542 | 14 | SRR372645 | 35,343,033 |
| 15 | SRR372644 | 33,870,711 | 15 | SRR372643 | 18,878,440 |
| 16 | SRR372642 | 33,437,124 | 16 | SRR372641 | 35,003,820 |
| 17 | SRR372640 | 35,133,221 | 17 | SRR372639 | 31,413,165 |
| 18 | SRR372638 | 28,670,513 | 18 | SRR372637 | 32,131,568 |
| 19 | SRR372636 | 30,822,280 | 19 | SRR372635 | 32,458,345 |
| 20 | SRR372634 | 25,672,582 | 20 | SRR372633 | 16,507,199 |
| | | | 21 | SRR372629 | 20,421,312 |
| **Hepatocellular Carcinorma** | | | | | |
| **Normal Samples** | | | **Tumour Samples** | | |
| **Sl No** | **SRR list** | **Total Number of Reads** | **Sl No** | **SRR list** | **Total Number of Reads** |
| 1 | SRR1642941 | 16,603,341 | 1 | SRR1642942 | 16,717,834 |
| 2 | SRR1642943 | 17,792,026 | 2 | SRR1642946 | 15,989,919 |
| 3 | SRR1642945 | 17,199,935 | 3 | SRR1642948 | 19,656,886 |
| 4 | SRR1642949 | 21,226,581 | 4 | SRR1642950 | 20,922,731 |
| 5 | SRR1642951 | 23,625,569 | 5 | SRR1642952 | 28,312,100 |
| 6 | SRR1642953 | 9,954,488 | 6 | SRR1642954 | 11,923,774 |
| 7 | SRR1642955 | 13,051,854 | 7 | SRR1642956 | 10,226,512 |
| 8 | SRR1642957 | 9,170,685 | 8 | SRR1642958 | 9,302,762 |
| 9 | SRR1642959 | 11,348,075 | 9 | SRR1642960 | 11,297,425 |
| 10 | SRR1642961 | 10,776,584 | 10 | SRR1642962 | 10,330,569 |
| 11 | SRR1642963 | 11,279,698 | 11 | SRR1642964 | 9,241,593 |
| 12 | SRR1642965 | 11,067,843 | 12 | SRR1642966 | 12,934,901 |
| 13 | SRR1642967 | 13,231,006 | 13 | SRR1642968 | 13,555,600 |

| | | | | | |
|---|---|---|---|---|---|
| 14 | SRR1642969 | 10,721,275 | 14 | SRR1642970 | 10,163,737 |
| 15 | SRR1642971 | 10,550,802 | 15 | SRR1642972 | 11,090,706 |
| 16 | SRR1642973 | 12,966,238 | 16 | SRR1642974 | 11,892,742 |
| 17 | SRR1642975 | 13,613,148 | 17 | SRR1642976 | 11,607,362 |
| 18 | SRR1642977 | 22,315,149 | 18 | SRR1642978 | 10,707,105 |
| 19 | SRR1642979 | 10,627,811 | 19 | SRR1642980 | 8,413,826 |
| 20 | SRR1642981 | 11,498,149 | 20 | SRR1642982 | 11,074,809 |
| 21 | SRR1642983 | 12,045,606 | 21 | SRR1642984 | 9,696,047 |
| 22 | SRR1642985 | 11,820,167 | 22 | SRR1642986 | 11,415,996 |
| 23 | SRR1642987 | 12301261 | 23 | SRR1642988 | 10,964,981 |

| **Carcinomas of the bladder** | | | | | |
|---|---|---|---|---|---|
| **Normal Samples** | | | **Tumour Samples** | | |
| **Sl No** | **SRR list** | **Total Number of Reads** | **Sl No** | **SRR list** | **Total Number of Reads** |
| 1 | SRR333656 | 13,964,069 | 1 | SRR333655 | 18,900,508 |
| 2 | SRR333658 | 18,961,597 | 2 | SRR333657 | 17,550,728 |
| 3 | SRR333660 | 15,606,203 | 3 | SRR333659 | 19,114,398 |
| 4 | SRR333662 | 16,012,177 | 4 | SRR333661 | 18,672,525 |
| 5 | SRR333664 | 14,333,941 | 5 | SRR333663 | 17,840,448 |
| 6 | SRR333666 | 11,350,981 | 6 | SRR333665 | 9,709,277 |
| 7 | SRR333668 | 17,755,521 | 7 | SRR333667 | 18,493,909 |
| 8 | SRR333670 | 10,019,292 | 8 | SRR333669 | 13,103,308 |
| 9 | SRR333672 | 17,654,215 | 9 | SRR333671 | 20,072,244 |
| 10 | SRR333674 | 19,283,954 | 10 | SRR333673 | 18,695,794 |
