# Supplementary material for: An approach to forecast human cancer by profiling microRNA expressions from NGS data
Source: BMC Cancer. 2017 Jan 25;17:77. doi: 10.1186/s12885-016-3042-2 (PMC5267436; doi:10.1186/s12885-016-3042-2)
Supplement: Additional file 2 — List of disease specific microRNAs used in the study. (PDF 30 kb) [file 12885_2016_3042_MOESM2_ESM.pdf]

Additional File 2: List of disease specific microRNAs with respect to Lung Cancer, Hepatocellular Carcinoma and Carcinomas of the bladder used in the current study

SL. No	Lung Cancer	Hepatocellular Carcinoma	Carcinomas of bladder
1	hsa-let-7c-5p	hsa-let-7c-5p	hsa-miR-16-5p
2	hsa-let-7d-5p	hsa-let-7d-5p	hsa-miR-17-5p
3	hsa-let-7e-5p	hsa-let-7e-5p	hsa-miR-18a-5p
4	hsa-let-7g-5p	hsa-miR-18a-5p	hsa-miR-19a-3p
5	hsa-let-7i-5p	hsa-miR-23a-3p	hsa-miR-23a-3p
6	hsa-miR-7-1-3p	hsa-miR-27a-3p	hsa-miR-26a-5p
7	hsa-miR-17-5p	hsa-miR-29a-3p	hsa-miR-26b-5p
8	hsa-miR-18a-5p	hsa-miR-30a-5p	hsa-miR-96-5p
9	hsa-miR-18a-3p	hsa-miR-100-5p	hsa-miR-99a-5p
10	hsa-miR-19a-3p	hsa-miR-16-2-3p	hsa-miR-103a-3p
11	hsa-miR-19b-3p	hsa-miR-197-3p	hsa-miR-192-5p
12	hsa-miR-20b-5p	hsa-miR-148a-3p	hsa-miR-10b-5p
13	hsa-miR-24-1-5p	hsa-miR-30d-5p	hsa-miR-34a-5p
14	hsa-miR-29a-3p	hsa-miR-34a-5p	hsa-miR-182-5p
15	hsa-miR-30c-2-3p	hsa-miR-182-5p	hsa-miR-183-5p
16	hsa-miR-33a-5p	hsa-miR-183-5p	hsa-miR-205-5p
17	hsa-miR-92b-3p	hsa-miR-199b-5p	hsa-miR-210-3p
18	hsa-miR-1-3p	hsa-miR-199b-3p	hsa-miR-214-3p
19	hsa-miR-23a-3p	hsa-miR-204-5p	hsa-miR-221-5p
20	hsa-miR-101-3p	hsa-miR-210-3p	hsa-miR-221-3p
21	hsa-miR-103a-3p	hsa-miR-217	hsa-miR-222-3p
22	hsa-miR-197-3p	hsa-let-7g-5p	hsa-miR-223-3p
23	hsa-miR-135b-5p	hsa-let-7i-5p	hsa-miR-200b-3p
24	hsa-miR-138-5p	hsa-miR-30b-5p	hsa-miR-23b-3p
25	hsa-miR-146a-5p	hsa-miR-143-5p	hsa-miR-125b-5p
26	hsa-miR-148a-3p	hsa-miR-143-3p	hsa-miR-133a-3p
27	hsa-miR-153-3p	hsa-miR-145-5p	hsa-miR-141-3p
28	hsa-miR-186-5p	hsa-miR-125a-5p	hsa-miR-143-3p
29	hsa-miR-187-3p	hsa-miR-136-3p	hsa-miR-144-5p
30	hsa-miR-194-5p	hsa-miR-146a-5p	hsa-miR-145-5p
31	hsa-miR-199b-3p	hsa-miR-185-5p	hsa-miR-126-5p
32	hsa-miR-200a-3p	hsa-miR-186-5p	hsa-miR-126-3p
33	hsa-miR-210-3p	hsa-miR-200a-3p	hsa-miR-185-5p
34	hsa-miR-215-5p	hsa-miR-151a-5p	hsa-miR-195-5p
35	hsa-miR-21-5p	hsa-miR-151a-3p	hsa-miR-200c-3p
36		hsa-miR-92b-3p	hsa-miR-155-5p
37		hsa-miR-151b	hsa-miR-29c-3p
38		hsa-miR-21-5p	hsa-miR-200a-3p
39		hsa-miR-17-5p	hsa-miR-335-5p
40		hsa-miR-122-5p	hsa-miR-429
41		hsa-miR-101-3p	hsa-miR-451a
42		hsa-miR-125b-5p	hsa-miR-409-3p
43			hsa-miR-146b-5p
44			hsa-miR-493-5p
45			hsa-miR-320c
