# Supplementary material for: An approach to forecast human cancer by profiling microRNA expressions from NGS data
Source: BMC Cancer. 2017 Jan 25;17:77. doi: 10.1186/s12885-016-3042-2 (PMC5267436; doi:10.1186/s12885-016-3042-2)
Supplement: Additional file 3 — Pre-processed NGS data samples with respect to normal and tumour tissues used in the study. (PDF 43 kb) [file 12885_2016_3042_MOESM3_ESM.pdf]

Additional File 3: Preprocessed NGS data samples of Normal and Tumour tissues with respect to Lung Cancer, Hepatocellular Carcinorma and Carcinomas of the bladder

Lung Cancer							
Normal Samples				Tumour Samples			
Sl No	SRR list	Total Number of Reads	Total Number of quality reads	Sl No	SRR list	Total Number of Reads	Total Number of quality reads
1	SRR372672	53,022,826	40,456,567	1	SRR372671	41,195,137	37,527,724
2	SRR372670	48,415,574	37,881,724	2	SRR372669	34,754,806	25,858,278
3	SRR372668	38,287,217	35,288,346	3	SRR372667	37,776,488	33,503,199
4	SRR372666	35,143,921	33,572,344	4	SRR372665	37,507,931	35,582,584
5	SRR372664	33,656,348	30,399,221	5	SRR372663	33,544,033	30,515,856
6	SRR372662	32,770,003	28,866,319	6	SRR372661	37,053,587	35,709,833
7	SRR372660	37,049,180	31,857,768	7	SRR372659	37,140,785	32,634,806
8	SRR372658	33,354,253	27,750,442	8	SRR372657	39,753,719	36,722,436
9	SRR372656	36,446,549	34,294,229	9	SRR372655	33,783,427	31,307,401
10	SRR372654	33,777,251	31,335,908	10	SRR372653	32,579,770	30,100,982
11	SRR372652	25,425,497	19,821,127	11	SRR372651	37,486,200	34,550,722
12	SRR372650	25,709,897	22,732,035	12	SRR372649	36,416,257	31,554,431
13	SRR372648	35,058,620	31,000,768	13	SRR372647	36,647,675	31,299,960
14	SRR372646	35,759,542	31,389,437	14	SRR372645	35,343,033	31,713,572
15	SRR372644	33,870,711	30,378,391	15	SRR372643	18,878,440	14,662,460
16	SRR372642	33,437,124	33,316,351	16	SRR372641	35,003,820	34,792,286
17	SRR372640	35,133,221	34,813,137	17	SRR372639	31,413,165	31,194,966
18	SRR372638	28,670,513	27,800,828	18	SRR372637	32,131,568	31,841,130
19	SRR372636	30,822,280	30,230,479	19	SRR372635	32,458,345	32,140,731
20	SRR372634	25,672,582	24,098,484	20	SRR372633	16,507,199	14,819,796
				21	SRR372629	20,421,312	18,511,100
Hepatocellular Carcinorma							
Normal Samples				Tumour Samples			
Sl No	Run	Total Number of Reads	Total Number of quality reads	Sl No	Run	Total Number of Reads	Total Number of quality reads
1	SRR1642941	16,603,341	16,602,920	1	SRR1642942	16,717,834	12,970,625
2	SRR1642943	17,792,026	17,791,488	2	SRR1642946	15,989,919	15,989,541
3	SRR1642945	17,199,935	17,199,507	3	SRR1642948	19,656,886	19,656,445
4	SRR1642949	21,226,581	21,225,970	4	SRR1642950	20,922,731	20,922,227
5	SRR1642951	23,625,569	23,625,014	5	SRR1642952	28,312,100	28,311,416
6	SRR1642953	9,954,488	9,954,352	6	SRR1642954	11,923,774	11,923,639
7	SRR1642955	13,051,854	13,051,638	7	SRR1642956	10,226,512	10,226,364
8	SRR1642957	9,170,685	9,170,541	8	SRR1642958	9,302,762	9,302,618
9	SRR1642959	11,348,075	11,347,922	9	SRR1642960	11,297,425	11,297,279
10	SRR1642961	10,776,584	10,776,447	10	SRR1642962	10,330,569	10,330,427

11	SRR1642963	11,279,698	11,279,544	11	SRR1642964	9,241,593	9,241,481
12	SRR1642965	11,067,843	11,067,621	12	SRR1642966	12,934,901	12,934,608
13	SRR1642967	13,231,006	13,230,731	13	SRR1642968	13,555,600	13,555,353
14	SRR1642969	10,721,275	10,721,015	14	SRR1642970	10,163,737	10,163,553
15	SRR1642971	10,550,802	10,550,583	15	SRR1642972	11,090,706	11,090,484
16	SRR1642973	12,966,238	12,965,931	16	SRR1642974	11,892,742	11,892,521
17	SRR1642975	13,613,148	13,612,886	17	SRR1642976	11,607,362	11,607,182
18	SRR1642977	22,315,149	22,314,762	18	SRR1642978	10,707,105	10,706,925
19	SRR1642979	10,627,811	10,627,630	19	SRR1642980	8,413,826	8,413,693
20	SRR1642981	11,498,149	11,497,979	20	SRR1642982	11,074,809	11,074,621
21	SRR1642983	12,045,606	12,045,390	21	SRR1642984	9,696,047	9,695,900
22	SRR1642985	11,820,167	11,819,931	22	SRR1642986	11,415,996	11,415,766
23	SRR1642987	12301261	12,301,051	23	SRR1642988	10,964,981	10,964,825
**Bladder Cancer**							
**Normal Samples**				**Tumour Samples**			
**Sl No**	**Run**	**Total Number of Reads**	**Total Number of quality reads**	**Sl No**	**Run**	**Total Number of Reads**	**Total Number of quality reads**
1	SRR333656	13,964,069	2,958,024	1	SRR333655	18,900,508	687,649
2	SRR333658	18,961,597	937,364	2	SRR333657	17,550,728	1,325,561
3	SRR333660	15,606,203	2,888,676	3	SRR333659	19,114,398	694,480
4	SRR333662	16,012,177	2,877,239	4	SRR333661	18,672,525	842,369
5	SRR333664	14,333,941	3,229,522	5	SRR333663	17,840,448	1,475,901
6	SRR333666	11,350,981	3,268,990	6	SRR333665	9,709,277	178,510
7	SRR333668	17,755,521	2,288,703	7	SRR333667	18,493,909	1,177,372
8	SRR333670	10,019,292	2,906,254	8	SRR333669	13,103,308	459,829
9	SRR333672	17,654,215	2,239,830	9	SRR333671	20,072,244	493,537
10	SRR333674	19,283,954	1,079,859	10	SRR333673	18,695,794	493,537
