# Supplementary material for: An approach to forecast human cancer by profiling microRNA expressions from NGS data
Source: BMC Cancer. 2017 Jan 25;17:77. doi: 10.1186/s12885-016-3042-2 (PMC5267436; doi:10.1186/s12885-016-3042-2)
Supplement: Additional file 4 — Normalized expression values of specific set of microRNAs associated with the lung cancer. (PDF 75 kb) [file 12885_2016_3042_MOESM4_ESM.pdf]

Additional File 4: Normalized expression values of microRNAs associated with Lung Cancer (page 1)

sl No.	hsa-let-7c-5p	hsa-let-7d-5p	hsa-let-7e-5p	hsa-let-7g-5p	hsa-let-7i-5p	hsa-miR-7-1-3p	hsa-miR-17-5p	hsa-miR-18a-5p	hsa-miR-18a-3p	hsa-miR-19a-3p	hsa-miR-19b-3p	hsa-miR-20b-5p	hsa-miR-24-1-5p
1	0.798812496	2.390591713	0.320442815	2.229339503	0.848099386	1.219199775	-0.546800096	-0.454917199	-0.426249074	-0.613762685	-0.393620865	-0.412642077	3.717940386
2	0.183872482	1.707537459	-0.063152994	2.404196614	0.518341691	0.062583243	-0.541218529	-0.664443228	-0.43517971	-0.904917769	-1.187051797	-0.547271919	0.158067694
3	0.33913031	0.186589385	1.290954365	0.275155231	0.2514736	-0.889765033	-0.568378536	-0.239308702	-0.281230991	-0.278389265	-0.546806518	3.602079164	-0.452169539
4	2.147597445	0.147904624	1.749309756	-0.200515995	0.325445737	-0.117684605	0.865426103	0.713792776	0.445960559	0.784751989	0.445783966	2.029764406	0.197409568
5	0.790930173	0.052843214	-0.274159695	-0.237525472	-0.615753228	-0.664475667	-0.48600742	-0.158848449	-0.354265912	-0.223018807	-0.401064556	-0.059882164	-0.41905799
6	-0.139819958	0.125424954	0.991760963	-0.348100379	-0.280540867	-0.110416103	0.169059948	0.050171986	-0.599642576	0.071792446	0.335959909	0.09018267	0.161916418
7	-0.438566153	-0.638543457	-0.303443078	-0.569242506	3.606037639	0.12208442	1.696491502	-0.111006862	0.54479904	-0.127911107	1.523677154	-0.233708681	-0.099693076
8	-0.058643779	-0.373517066	-1.291085406	0.554179658	-0.282463535	-0.087810023	-0.677479108	-0.55219298	-0.30724799	-0.766276208	-0.522551578	-0.243515163	-0.150945709
9	-0.812378905	-0.814505564	0.802961162	-0.832288548	-0.420276954	1.653555171	3.334923502	4.119857018	4.131478527	3.918316944	3.484238838	-0.405840178	1.166342713
10	1.431305392	-0.426152594	0.04773413	-0.239654003	-0.693871452	-0.994308007	-0.578744111	-0.356179207	-0.21657218	-0.402385891	-0.463784183	-0.336571496	-0.962018931
11	-0.217429377	0.515262136	0.350856301	-0.100672657	0.549929131	0.147763215	0.252868898	0.177859915	0.175597681	0.067257667	-0.053329138	-0.467271713	0.073008435
12	-1.004970417	-0.917919821	-0.753789078	-0.531443234	-0.407053431	-0.098799319	-0.438978943	-0.5576944	-0.560414724	-0.577745412	-0.76836402	-0.095561532	-0.429937325
13	2.154362077	2.227339775	2.225443581	1.243769683	0.262403474	0.410830461	-0.183527034	-0.315294203	-0.191988201	-0.493801679	-0.204983466	-0.559583068	0.891983987
14	0.606452814	-0.432630385	-0.559889289	0.413660722	-0.487113343	-1.24838473	0.264202821	0.141167885	-0.221164257	0.494970018	0.480579836	-0.395926466	-0.333468234
15	-0.79792742	-0.866038588	0.199754983	-0.511033002	0.448996047	-1.052603663	0.508057924	-0.300435278	-0.17681943	-0.018709938	-0.24351676	-0.590345855	-0.703033085
16	-0.612377752	-0.875797514	-0.772196341	-1.47521755	-1.099927461	-0.68294059	-0.40347817	-0.464217727	-0.522331512	-0.490098441	-0.663873281	-0.155297372	-0.825728668
17	-0.589463452	-0.306595629	-0.636499787	0.516159693	0.343158805	-0.132212315	-0.432446802	0.153040273	-0.053081177	0.53031755	0.170176059	0.450948938	-0.761435002
18	-0.861009422	-0.10734126	0.064520983	0.186953831	-0.346532049	0.407676569	-0.146789723	-0.061936964	0.289547899	0.038192734	0.253720394	-0.241475298	-0.631258654
19	-0.844839167	-0.809624619	-1.276193225	-1.086474442	-0.709134003	-1.416235561	-1.131589563	-0.494383348	-0.516569108	-0.574592464	-0.896636331	-0.55438181	-0.251943858
20	-1.132299506	0.335451546	-0.409628053	-0.517705944	-0.709397387	2.724671388	0.047032812	-0.083428678	-0.299669442	0.068319109	0.267647227	-0.362670179	-0.254925681
21	-0.942737883	-1.120278311	-1.703702093	-1.173541203	-1.101821799	0.747271372	-1.002625475	-0.541602628	-0.424957424	-0.50230879	-0.616200891	-0.511030205	-0.091053447
23	1.133809976	0.804432418	0.261869221	2.691399588	3.11058928	2.231744328	-0.223869993	-0.75208719	1.437843065	-1.103111066	-0.086397777	-0.33829018	-0.13009134
24	0.119760995	-0.588696089	-0.375290766	-0.093030184	-0.208719891	1.112764329	-0.154402338	-0.252749129	-0.338674156	0.232167349	0.622406432	0.144060924	-0.339836669
25	0.931467462	0.425581805	1.409368508	0.781745678	1.043723298	0.666986637	-0.017303882	2.583356015	1.097057741	2.651922076	2.173607688	1.584223993	-0.056897574
26	2.342722963	-0.420053775	0.619837853	-0.516912173	-0.382379858	-0.650944427	-0.314307572	-0.568604281	-1.326755761	-0.521248846	-0.656803366	-0.646006684	-0.367709573
27	0.324394053	-0.408270581	0.160371911	-0.027866937	0.171596471	0.222226675	-0.267666387	0.126678645	-0.064951081	0.490234644	0.527206756	0.694142589	-0.445218295
28	1.780360909	1.054409198	1.452570167	2.50006345	1.68747488	0.486322663	0.040459323	-0.767826172	-0.265168496	-1.31960976	-0.996611401	-0.482164705	-0.000404163
29	-1.349560478	1.237465155	-0.865759356	0.457407538	0.406190485	-0.160814169	0.314017955	-0.406577917	0.972464082	-1.425433251	-1.087797389	0.354780197	0.208068762
30	-0.262190467	-0.453400464	-0.056938482	-0.280546379	-0.162993801	0.501028207	-0.292990707	0.163838714	0.238519372	0.649800816	1.504133489	-0.495536386	-0.146790202
31	0.208916544	0.040723387	0.744523486	0.364702333	0.410171728	0.003120582	-0.070798756	-0.11184069	1.064582007	-0.215530941	0.060795015	-1.307660237	-0.291770381
32	0.062594227	-0.253808606	0.058621781	-0.493617489	-0.288835444	-0.485031313	-0.489344731	-1.020344518	0.917525836	-1.040113476	-0.941554539	-1.327081702	-0.207343949
33	-0.732552057	-0.586635516	-0.754138388	-0.456368696	-0.712261067	-1.141142159	-0.491631727	-0.98875817	-1.107638591	-0.824827521	-1.049148335	-0.200321981	-0.356493537
34	-0.467894592	-0.659517495	-0.58992678	-0.605708423	-0.324385061	-0.580862129	-0.442918218	-0.98137119	-0.969592674	-0.566121547	-0.468150891	-1.375323259	-0.343489214
35	-0.628330331	-0.443067795	-0.292392398	-0.176407722	-0.297152337	-0.576743761	-0.359658285	-0.406830637	-0.408153244	-0.176477599	-0.56504254	-1.084423764	-0.357129896
36	-0.378474036	-0.434013568	-0.226662912	-0.0806179	-0.317024936	-0.192013222	-0.201860726	0.165914772	-0.688273122	0.4449189	0.151767195	0.438325515	-0.305359386
37	-0.606941368	-0.348313487	0.251723257	-0.11013461	-0.007799124	-0.466675781	-0.193695615	0.542652055	0.154687542	1.25857764	1.330671611	0.010496888	-0.271320113
38	-0.553890048	-0.819655083	-1.231224528	-0.709717534	-0.678189681	-1.032882823	-0.373603571	0.059608766	-0.362953372	0.124379752	-0.199295423	1.827224416	-0.455068824
39	-0.652005953	-0.63623185	-0.484901117	-0.521658657	-0.532979201	-0.22090789	-0.407032813	-0.102153124	-0.321788719	-0.480599769	-0.680723076	-0.707625312	-0.228699736
40	-1.320806059	-0.653997517	-1.814842822	-0.998535553	-1.354589864	0.4097571	-0.139352535	2.159110818	1.546280678	1.273425168	1.223258815	1.217826862	-0.220147599
41	-0.973693381	-0.277597085	-0.762852799	-0.675723196	-1.03014066	1.885885851	-0.07916958	1.342574288	0.644971983	0.153830455	0.578275927	0.032831028	0.135724639

Normalized Expression values of microRNAs associated with Lung Cancer (page 2)

sl No.	hsa-miR-29a-3p	hsa-miR-30c-2-3p	hsa-miR-33a-5p	hsa-miR-92b-3p	hsa-miR-1-3p	hsa-miR-23a-3p	hsa-miR-101-3p	hsa-miR-103a-3p	hsa-miR-197-3p	hsa-miR-135b-5p	hsa-miR-138-5p	hsa-miR-146a-5p
1	-1.112560133	-1.019873686	-0.237167515	0.821124304	0.055546898	0.542789883	-0.622756764	-1.053955861	-0.031402837	1.340486754	-0.536256292	-1.168414854
2	-1.537676744	-0.531691279	-0.560733236	-0.564995034	1.063766079	0.687076786	-0.273899669	-0.753381806	-1.875700345	0.071428677	-0.830001294	-1.307049783
3	0.159494922	-0.408877578	-0.393260368	1.97683621	-0.47623079	0.534876128	-0.875744582	-0.306861033	1.358555079	0.605288094	-0.502607713	-0.108824417
4	0.173076768	-0.680595355	-0.159885993	0.729533512	0.211863693	-0.537982009	-0.621493652	-0.116825094	0.942071433	-0.661967229	2.134188345	1.964707925
5	0.23985443	2.761319309	-0.647515529	1.30915348	0.407544021	0.333298148	1.34076651	-0.351714428	1.04598292	-1.137482974	-0.250228549	1.317688173
6	1.69194443	-0.047588608	-0.888706175	0.422246521	-0.162186464	0.65390693	-0.035956404	0.057878777	0.127894596	-0.676999265	-0.656359036	0.096738725
7	-1.120320245	0.269580187	2.207958795	-0.038078681	-1.001226044	-0.377480832	-0.114006969	-1.012613995	-0.814139843	0.072286355	2.621055156	-0.414571834
8	-1.205563732	1.405565319	-0.696422272	0.475386976	2.182176398	1.009864088	2.641854416	-0.91294063	-0.567561404	-1.195062376	-0.342569309	-1.275637951
9	-0.095862257	-0.994298668	1.779135374	-0.37943795	-1.179804284	-0.259772714	-0.917310674	1.322838564	0.979218816	-0.951856332	-0.728038388	-0.52305249
10	0.780546121	0.964057821	-0.491850311	-0.735290053	0.254568148	-1.240640327	0.40577618	-0.29712647	-0.281051286	-0.581850599	1.573457549	0.070794044
11	0.105841108	0.638915224	0.008794278	1.162886025	-0.555289375	1.637062294	-1.078156176	0.212973568	2.149802418	-0.682065035	1.189096648	0.324058137
12	-0.103293412	0.570921343	0.014413934	1.29884837	0.731071302	-0.243575769	1.047351008	1.569172793	-0.235863737	1.382679685	-0.664200425	-0.384549785
13	-1.440553542	-0.095857643	2.600419216	-0.715626387	2.224975796	1.052707634	-0.580284436	1.217184045	0.700637854	-0.411397509	-0.603035155	-1.263795825
14	1.173458491	-0.424983426	0.225327335	-1.392952484	0.981173598	-0.905451093	-0.090056427	-0.372106945	-1.188152526	0.382184527	0.006017639	-0.122100647
15	-1.003729117	-0.73808177	0.408714028	-1.434430064	-0.574448693	-0.687609043	0.134939844	2.672381419	-1.161778196	2.206881733	-0.877715976	-0.386216172
16	-0.78755616	1.435208435	-1.014067118	-0.530116629	0.098536014	-0.808548638	0.455913946	0.178082274	-0.334531894	-0.986553316	0.09681932	-0.748078111
17	1.413790189	-0.789821772	-0.667969335	-1.448804181	-0.690545439	-0.509422533	0.082892422	-0.726303248	-0.010015806	-1.231988875	-0.308669589	1.289033108
18	0.716741821	-0.423575037	0.188494723	-0.805390438	-1.069387523	-0.772366772	-0.308580318	-0.660117857	0.474849044	0.955455798	0.005592821	1.539032868
19	-0.164345329	-0.954363615	-0.404344116	-0.862665535	-1.110552122	-1.590849189	-0.895845347	-0.859506947	-0.96658152	0.671678973	-0.420334951	1.300920167
20	1.385206355	-1.089547017	-0.708949883	0.050401356	-1.058018618	2.333387857	-1.397737572	0.923532326	0.669782157	-0.360801459	-0.297413284	0.575901429
21	0.731506036	0.153587817	-0.562385833	0.661370681	-0.333532595	-0.851270829	1.702334664	-0.730589452	-0.982014924	1.189654372	-0.608797516	-0.776582707
23	-1.242623937	-0.78800319	-0.220086026	0.154389453	1.847651212	2.950985903	0.749926127	-1.041830383	-0.297092876	0.299281066	-0.355754101	-1.105614476
24	1.001519036	1.13214768	-0.299249095	2.380836193	2.498219985	-0.097130612	1.674287727	0.127120811	-0.256274999	0.013207237	-0.238801829	0.400739261
25	2.321842361	1.161406677	-0.229697242	1.476667758	0.329400136	0.253213565	0.87741397	-0.320581672	0.97137774	0.19129915	0.243822526	2.099714126
26	0.496600689	0.199131489	-0.338757842	1.685340526	2.056076756	-0.424069168	-0.061575366	-0.678233756	-0.663314001	-0.885018576	-0.328909975	0.102510871
27	0.153056994	-0.652939519	-0.321520088	-0.885256952	-0.823856782	-0.563783795	-0.076936062	0.853935145	-0.403913804	0.184193123	0.168462388	1.452695286
28	-0.782024325	1.902628806	-0.176417205	-0.21011361	-0.077117025	2.018116343	-0.388017532	2.179524332	-0.44063507	-0.769845806	-0.136608809	-1.03661067
29	-2.152333104	-2.046071018	0.322954539	-1.204232721	-1.01368098	-0.277085629	-2.139304495	-2.300577865	-1.281239639	-1.493074416	-0.684380785	-1.122920046
30	1.165666653	-0.062945185	-0.159560925	0.09291178	-0.21723317	-0.059974862	1.127159447	-0.116039333	-0.201700602	0.582079323	-0.247177873	-0.066071623
31	-0.288070077	1.03476597	-0.248491701	-0.153576712	-0.552270351	-0.103395234	-0.780540914	0.034130722	0.553713958	0.755946384	0.237216187	-0.256855939
32	-1.015958383	-0.143701663	-0.32093097	-0.531411197	-0.594785302	-0.565728768	-1.800721023	-1.332561202	0.109075229	-0.840554398	-0.445097254	-0.663938516
33	-0.390379864	0.001248576	-0.308819201	-0.875271396	-0.526841715	-0.757601774	-1.000955268	-0.958582717	-0.566113289	-1.31086184	-0.257955271	-0.761819745
34	0.105700471	-0.360383473	-0.202071058	-0.079496117	0.061686792	0.427445162	0.573223541	0.112966221	-0.331248558	0.543631331	-0.296097036	-0.663297483
35	0.112198185	0.138173341	-0.187150204	-0.917426275	-0.214383314	-0.427993061	0.10251089	0.542872967	-0.425016931	0.209975461	-0.334932744	-0.215716454
36	0.274867843	0.554054952	-0.269957353	0.185968719	0.49187409	-0.529418451	0.504550103	0.757147968	-0.425420627	-0.622683157	-0.149690789	-0.235076932
37	0.442086641	0.787726197	-0.207618987	-0.471609198	-0.294361979	-0.70607083	0.669124825	0.011390908	-0.161670947	0.405070398	-0.371826994	0.793088986
38	-0.648745523	-1.028318345	-0.363449674	-0.808168332	-0.630285344	-0.89957523	-0.513739402	-0.612131317	-0.60128562	-0.917181717	-0.481226934	-0.058098813
39	0.283516702	0.460404847	-0.252805076	-0.407175938	-0.791203735	-0.245897859	-0.253641687	0.734778209	-0.230545011	-0.099878361	-0.317544782	0.244708032
40	0.087135362	-0.716242603	-0.233034656	0.896790532	-0.770046494	0.022260396	0.4453385	0.27246806	0.563688741	0.453558568	-0.094570135	-0.625044685
41	1.066306019	0.282223442	-0.189529074	0.87122927	-0.301449919	1.143410608	1.239749231	1.357401442	0.431399232	0.149894842	-0.037852478	-0.651258691

Additional File 4:   Normalized Expression values of microRNAs associated with Lung Cancer (page 3)

sl No.	hsa-miR-148a-3p	hsa-miR-153-3p	hsa-miR-186-5p	hsa-miR-187-3p	hsa-miR-194-5p	hsa-miR-199b-3p	hsa-miR-200a-3p	hsa-miR-210-3p	hsa-miR-215-5p	hsa-miR-21-5p	class
1	-0.370505652	0.770326026	0.073080614	-0.360075992	-0.286735917	0.902799997	-0.155925997	-0.748157192	-0.204719275	-0.408091815	LT
2	0.034633969	0.4929166	-1.56851209	-0.54741329	-0.294286658	0.073549556	-0.452595542	-1.200453377	-0.19758667	-0.004876541	LT
3	0.033335939	0.123245969	-0.173481398	1.293846446	-0.293471398	1.995147595	-0.112868245	0.00808047	-0.205727759	0.254581654	LT
4	-0.463463296	-0.639781954	1.855235849	-0.268528108	-0.226397518	0.113525649	0.471398614	1.692171864	-0.213835264	0.089115111	LT
5	-0.608744033	-0.699624518	0.482860659	-0.21161262	-0.025222232	-0.667286761	-0.325915893	-0.730859641	0.035347549	-0.98252079	LT
6	-0.746239052	-0.985852278	0.21503894	-0.52955615	-0.180094468	0.313708264	0.397885946	0.495469752	-0.257277431	2.189204589	LT
7	1.146325418	-0.632473301	-1.109257816	1.180043173	-0.296589054	-0.796583219	-0.321559566	-0.02443012	-0.238626275	-0.717293452	LT
8	-0.482309299	-0.302688154	-1.265844403	-0.494796887	-0.300839332	0.100664938	-0.722736598	-1.189487265	-0.229748017	-0.705357518	LT
9	-0.430198774	1.896755656	1.559517355	-0.049175015	-0.24957602	-0.630855687	4.114549877	1.58761492	-0.214668055	-0.588829282	LT
10	0.524497767	-0.941362905	-0.485059	-0.449198548	-0.280114899	-1.175868556	-0.21779766	-0.509377936	-0.271914649	-0.214988804	LT
11	-0.352443625	-0.575479517	1.020525596	-0.367986644	-0.276538985	2.453422978	-0.355015537	1.320975184	-0.211432774	0.635844885	LT
12	-0.527303839	-0.036174665	-0.965763706	3.713012703	-0.287843108	-0.710093369	-0.356952216	1.411179384	-0.204626	-0.365516568	LT
13	-1.212159838	1.063088924	0.436320625	0.039092044	-0.272111905	0.11578643	0.120312436	-1.079426868	-0.214072256	-0.548071331	LT
14	-0.308093471	-0.509146819	0.063109675	-0.466636935	0.382809665	-0.692167756	0.406293046	-0.942607844	-0.265852404	-0.465545718	LT
15	-0.788811888	-0.636468039	-0.188895972	0.379305099	-0.17074884	0.870992587	-0.162057559	1.273130859	-0.246778779	1.310996258	LT
16	2.201454361	-0.867671103	-0.947579918	-0.44161867	-0.280879496	-1.089193841	-0.547979694	-0.843710064	-0.231834544	-0.752365826	LT
17	-0.255140951	-0.383251762	1.311439855	-0.523285639	-0.150436668	-0.735421428	-0.608337256	-0.875595205	-0.226912583	-0.582216922	LT
18	0.577636422	-0.474092391	0.490348089	-0.521318772	-0.255258705	-1.154356063	-0.291431584	-0.586906542	-0.261199123	-1.039538172	LT
19	2.897822749	1.30344917	-0.678171067	-0.436532151	-0.284596588	-0.643280696	-0.379965473	0.828038897	-0.269440629	-0.825429308	LT
20	-0.210052367	-0.608072989	1.110721858	-0.436569605	-0.284295556	0.519410582	-0.553848872	-0.285210047	-0.224917034	2.202840535	LT
21	-0.660240539	2.642358051	-1.235633743	-0.50099444	4.313227684	0.836098798	0.054547772	0.399560771	4.355821974	1.518059016	LT
23	0.487443381	0.977741563	-0.31703498	-0.95813756	-0.224801443	3.202831184	-0.230578072	-1.075674396	0.04633639	-1.555966679	LNT
24	0.443656485	0.493475079	-0.281680402	2.940855543	-0.192489998	0.970811821	0.659512242	0.765026874	-0.027041102	-1.127152163	LNT
25	-0.038307173	-0.197421395	0.274949875	1.064917613	1.386156612	0.067613177	0.933385847	1.674139089	-0.772721756	-1.078894556	LNT
26	-0.241032387	-0.806297844	-0.346791028	1.097673301	-0.438935658	0.02004412	2.616398993	0.40749931	0.413467844	0.158994992	LNT
27	-0.898649576	-1.069260384	-0.16902678	-0.468104637	-0.159128172	-0.337382244	-0.1748802	0.265761691	-0.857407795	0.18760394	LNT
28	1.844455032	-0.110134632	-0.009413827	-0.952396805	0.13639744	1.523318432	0.697970907	-0.903179842	0.424893833	-1.450815555	LNT
29	-1.189441849	-0.967881299	-0.683762375	-1.213432076	1.673294255	-0.664960778	-0.554753266	-1.453640157	0.604698793	1.918116404	LNT
30	0.434843416	0.128306579	-0.00353977	0.969729673	-0.441905083	-0.320187083	1.264966301	2.492689569	-0.768331953	-0.601115592	LNT
31	0.52545702	0.298562903	-0.180567919	-0.210493456	-0.70160081	-0.287925616	0.89553438	0.349204512	-0.514370541	0.275506257	LNT
32	-1.301033303	-0.691223184	-0.188022025	-0.744190361	-0.780096096	-0.689275817	-0.7140345	-0.085447512	-0.805338284	1.794331451	LNT
33	-1.780111623	-0.489251835	-0.363936346	-0.639487377	-0.657190434	-0.992187847	-0.883824399	-0.28402896	-0.761432634	1.492004472	LNT
34	0.300753679	0.476919353	-0.247887402	0.148210811	-0.963800206	0.534759621	0.480016653	0.546007402	0.166931583	-0.140730427	LNT
35	0.660365724	-0.749370791	-0.247408984	-0.568253761	-0.658212151	0.112218921	0.063819665	-0.322383971	-0.326148111	-0.026256362	LNT
36	-0.720570941	0.184355533	-0.256442601	0.173777808	-0.204203369	-0.24894494	-0.241905451	0.245520146	-0.516802694	-0.098201205	LNT
37	0.482505899	-0.128633114	-0.152982355	-0.552113402	-0.107490627	-0.605662003	0.333588209	0.719356426	-0.600128001	-0.293471121	LNT
38	-0.779348242	-0.842805953	-0.410481544	-0.860311956	-0.277548882	-0.330385061	-1.090833134	-0.782411394	-0.415431305	1.067069454	LNT
39	1.351245782	0.923352392	-0.182597257	0.170538779	-0.705990519	-0.616881323	-0.372597231	-0.476000207	0.0600582	0.132648525	LNT
40	-0.154972867	1.809543154	-0.369580163	-0.57974493	0.410320763	-0.194831361	-1.223509775	-0.553890694	-0.075994636	0.269664997	LNT
41	1.638242733	2.408644893	-0.032292608	0.77185358	-0.255378936	0.25457486	-1.117467039	0.122414321	1.187789312	-1.029794362	LNT
