# Supplementary material for: An approach to forecast human cancer by profiling microRNA expressions from NGS data
Source: BMC Cancer. 2017 Jan 25;17:77. doi: 10.1186/s12885-016-3042-2 (PMC5267436; doi:10.1186/s12885-016-3042-2)
Supplement: Additional file 5 — Normalized expression values of specific set of microRNAs obtained with the hepatocellular carcinoma. (PDF 93 kb) [file 12885_2016_3042_MOESM5_ESM.pdf]

Additional File 5 : Normalized expression values of microRNAs associated with hepatocellular carcinoma (page 1)

sl No.	hsa-let-7c-5p	hsa-let-7d-5p	hsa-let-7e-5p	hsa-miR-18a-5p	hsa-miR-23a-3p	hsa-miR-27a-3p	hsa-miR-29a-3p	hsa-miR-30a-5p	hsa-miR-100-5p	hsa-miR-16-2-3p	hsa-miR-197-3p	hsa-miR-148a-3p	hsa-miR-30d-5p
1	-1.123849874	-1.128343388	-0.612404743	-0.230745355	-0.577382881	-1.021345407	-1.237322708	-0.984008581	1.322353714	1.555042216	3.948078464	-0.733853891	1.157513371
2	-0.478249649	-1.243131257	-0.72023508	0.103927957	-0.90284509	-1.264398659	-0.611543303	-0.8853681	-1.249406182	0.755262181	-0.498096324	-0.040770931	1.206290468
3	0.037586167	-0.568681259	-0.070552225	-0.458478227	0.105223194	0.971339597	0.057053357	0.223834977	-0.638296541	-1.076690506	-0.593462605	-0.106676312	-0.806468927
4	0.600804261	-0.366730699	-0.505784581	-0.420768463	-0.603298663	-0.106139142	-0.307429844	-0.205129379	0.313989358	-0.522913724	-0.426653554	0.146813299	-0.111402435
5	-1.221029218	-1.059137316	-0.917958168	-0.201961313	-0.92968925	-1.183031034	-1.265724079	-0.499327046	-1.245568169	-1.536183049	-0.682397838	-0.578674092	-1.21994264
6	-1.221910947	0.547037859	-0.083407142	0.122679933	0.22401149	2.466272541	3.513696483	0.769695984	-1.103396956	-0.534493675	-0.4555711	-1.247653614	-0.239131602
7	-1.442887366	-0.985527458	0.574755207	4.244634036	-0.14113547	0.405559313	-0.832858084	-0.812008785	-0.971603458	1.067864285	1.585355661	-0.87392824	-0.106527482
8	0.770657113	0.630647108	1.665529799	-0.311376103	2.220995793	0.63994556	0.604198604	0.473475824	0.760358671	-0.080801917	0.131364585	-1.122508626	-0.842650059
9	0.095787432	-0.072957999	-0.146768713	-0.375309846	-0.358917575	0.485798457	-0.185601682	-0.080842137	-0.147015254	-0.704857212	-0.553104922	-0.175716304	-0.838172486
10	-0.227867959	-0.289940208	-0.858731158	-0.212764641	-0.889710875	-1.159108295	-0.123113769	-0.326682061	-0.56923519	1.170856691	-0.582620835	0.685757379	0.257641134
11	1.085376046	-0.033429415	-0.745259268	-0.44443923	-0.756628755	-1.297385296	-0.352636946	-0.619115793	1.491183979	0.09102947	-0.385139794	1.728134082	1.372353838
12	1.264714715	-0.394630051	-0.722604884	0.186862313	-0.567411462	1.1679208	-0.085574133	3.822452114	-0.340751467	1.674716609	-0.347451502	1.71425906	-0.407309443
13	0.819160359	0.407687945	1.231935304	-0.392397987	0.736298006	0.721410791	0.506332148	0.592724625	1.276646513	-0.749945934	-0.052211474	-1.051783887	-0.750592596
14	-0.860732087	-0.92221602	-0.100237515	0.011116719	-0.032197499	0.321888348	-0.492535895	0.150756997	-1.220971898	0.776259065	-0.564829888	0.522294688	-0.067436774
15	-0.391025184	1.665843857	0.025804489	1.173575471	0.029885968	1.058895805	-0.349225884	0.006891519	-0.497241616	0.544684744	-0.135874858	0.043859752	0.756353847
16	2.326033139	2.32035265	3.115025517	-0.489308189	2.749425531	0.315989393	-0.132038135	-0.350002951	2.168976639	1.274685384	0.557746723	-1.429863122	-0.614029342
17	0.069048187	-0.603540324	-0.539962411	-0.371605736	0.289645051	-0.145962156	0.605477478	1.004777685	-0.14877697	-1.029896846	-0.551725679	0.880279927	-0.915889938
18	1.317055292	-0.217924564	-0.854262766	-0.502425	-0.814995564	-1.357773908	0.535647336	-0.64038009	0.434677021	0.129877304	-0.512230378	1.442664554	0.014553896
19	-0.840728103	-0.543134833	-0.208229899	-0.412478244	-0.751493117	-1.059170332	-1.188072123	-0.707157698	-0.87701005	-1.556777393	0.098173081	-0.062685769	-0.559946579
20	-0.336891716	-0.321906736	-0.499944226	-0.520682978	-0.281875433	-0.418156545	-0.328365229	0.343924456	0.624865424	-0.013730666	-0.061619172	0.172855586	0.67480087
21	-0.853146433	0.090592227	-0.826789157	-0.29570106	-0.812357358	-0.201499362	0.689234206	-0.402453732	-0.136658362	-1.568340673	-0.112170072	1.809880071	-0.557895486
22	1.079764871	0.865632775	1.220073869	-0.454688074	1.536082649	-0.148079533	-0.231711154	-0.08993135	1.23166148	0.200609182	0.4501321	-0.933352004	-0.524026207
23	-0.467669046	2.223437108	0.580007749	0.252334017	0.52837131	0.807029062	1.212113356	-0.786126479	-0.478780685	0.133744463	-0.255690619	-0.789331606	3.121914574
24	1.17359675	1.219570072	1.758366749	-0.615704958	0.888438447	1.545860479	0.552463846	0.790655061	1.288876801	0.1907538	1.192332499	-0.516611767	1.817983975
25	-0.374750813	-0.659646201	-0.978787418	0.608821886	-1.219829506	-0.724544359	-0.744185049	-0.266958882	-1.344447811	0.271144567	-1.444902951	-0.103885291	-2.039324275
26	-0.730566681	-0.87841097	-0.744106254	0.327910593	-0.79176502	-1.137067148	-1.036673723	-1.01653697	-0.832466992	-0.893102706	-1.392199748	0.573389922	-1.02978841
27	0.729237477	0.135120287	0.757626286	-1.327445621	0.17031273	1.28355357	-0.750678586	-0.639012937	1.159053576	-0.270614649	1.235237256	-0.545415786	0.189652874
28	0.043668195	0.514194838	0.801845121	-0.248274292	0.593031826	0.447540102	1.011265124	-0.645121521	0.140995325	1.49526898	0.787129827	-0.737859228	-0.181344725
29	-0.592960633	-0.88638393	-1.000372395	-0.147415007	-0.880366796	-1.000676377	-0.521632262	0.151474389	-1.08123206	-1.309961217	-0.956613199	1.626467066	-0.039558673
30	-0.498203166	-0.617978669	-1.175199157	0.427221114	-1.399009067	-0.701349603	-2.104179958	-0.595942619	-1.626329846	-0.697239995	-1.670813189	-0.676692903	-2.057801763
31	-1.860641179	-1.49786954	-0.83918341	2.562952345	-0.679223268	-0.585229402	0.260437006	-0.511553738	-0.101166955	1.030447184	0.212250893	-0.015778479	0.278714632
32	0.082690293	0.083343271	0.12008203	-0.415248531	-0.181058821	0.393287029	-0.369795482	-0.772373328	0.901855888	-1.280923101	-0.822218971	-0.009217245	-0.1165337
33	-0.501309572	-1.089659168	-1.084869778	-0.438319944	-1.003801198	-1.251688799	-1.371009644	-0.141621704	-0.649779604	-1.328830353	-0.541552758	2.046923168	-0.372424103
34	-0.326974018	-0.442231525	-0.549241832	0.434913598	-0.577643481	2.074840912	0.001149919	3.157362169	-0.254234952	0.265471364	0.370155107	2.135883455	1.457810959
35	-0.204322704	-0.632597386	-0.1859548	-0.71598069	-0.089696921	0.288818286	-0.224942415	0.00937315	-0.405504348	-1.816095764	-0.21332041	-0.737212999	-0.675548878
36	-0.410962949	0.258306193	0.03670864	0.164510644	0.187522061	-0.126781164	1.044205927	-0.078934601	0.438546063	0.153254615	-0.691118022	-0.752656254	-0.036377979
37	-0.536440707	-0.673149827	-0.786197555	0.725013757	-0.45777357	-1.001321311	0.797042564	0.769568397	-1.041536174	-0.373426552	-0.614915447	1.541825259	0.199471467
38	-0.302490307	-0.032398064	-0.436317265	1.354217699	0.060794488	0.672051676	1.110956901	1.666034113	-0.205874349	0.609351031	-0.033185296	0.203349067	1.000812547
39	-0.733473665	-0.104782037	-0.22156893	1.897955077	-0.270612282	0.135017388	2.409780436	0.443409753	0.182512276	-0.602396531	0.095725852	-0.48031777	0.245651779
40	-0.194562491	-0.213179824	-0.28733574	0.074732123	0.133036476	0.071429521	-0.008917858	-0.468712137	-0.723445893	1.172683383	-0.695232586	-0.244278121	-0.086651395
41	2.376059725	2.596077212	1.76243685	-1.189882124	1.622801926	-0.344122176	0.731476804	0.925618699	2.064381182	1.626638943	2.136360926	-0.65096728	1.6227305
42	2.573425012	2.172368558	2.337426244	-0.965119229	2.643304964	1.909699011	0.58358007	-0.036648773	0.509353492	1.55023116	0.499662658	-1.256160868	-0.370313311
43	0.06451261	0.698764772	0.419746966	-0.961176776	0.926667209	-0.088333867	-0.030233991	-0.708266378	0.862511482	0.552113462	0.527117743	-0.718889164	1.052352497
44	-0.399956327	-0.359656691	-0.498657332	-0.836535381	-0.902459158	-0.947844959	-0.441896417	-1.070617637	-0.821532966	-0.001416393	0.905072313	0.409444779	-0.455382538
45	0.62442515	0.410198629	0.793552981	-0.717146284	1.227328962	-0.913138808	-0.898213215	-0.961194504	1.539465866	-0.343351227	1.115027503	-1.091339561	-0.404131479
46	-0.611137408	-1.011521932	-1.112339612	-2.043163591	-1.234117955	-1.176473971	0.18501654	-1.191242878	-0.901389793	-1.139807519	-0.222662107	1.114490531	0.288807497

Additional File 5 : Normalized expression values of microRNAs associated with hepatocellular carcinoma (page 2)

sl No.	hsa-miR-34a-5p	hsa-miR-182-5p	hsa-miR-183-5p	hsa-miR-199b-5p	hsa-miR-199b-3p	hsa-miR-204-5p	hsa-miR-210-3p	hsa-miR-217	hsa-let-7g-5p	hsa-let-7i-5p	hsa-miR-30b-5p
1	-0.670543015	-0.087152191	-0.142179923	-0.61403809	-0.71636429	0.176846817	1.574820255	1.603379156	-1.818953904	-1.048499028	0.56691225
2	-1.279234698	-0.336745138	-0.255033727	-0.585733736	-0.660911202	-0.475467806	-0.644307321	2.073498136	-1.617732686	-0.933561973	0.107783667
3	-0.440297145	-0.324098613	-0.323351671	-0.164702244	0.164048479	-0.471562885	-0.43970637	0.541541709	-0.427325056	-0.190579032	-0.676927941
4	1.297241606	-0.598768214	-0.58325441	-0.555336732	-0.549024404	0.624492961	-0.294218496	-0.606220326	-0.236037576	-0.555737244	-0.205631293
5	-0.312557148	0.03025018	0.023566224	-0.619762061	-0.733287392	-0.55305957	-0.170096662	-0.595138298	-1.600876368	-0.875208275	-1.418197484
6	0.754083406	-0.405171035	-0.382431897	-0.056171302	-0.112655817	-0.529137099	0.119103611	0.107320272	1.050622475	0.854554314	-0.116557369
7	-0.100714694	-0.061521986	-0.090986608	-0.238280671	-0.661841302	-0.591414814	2.74082736	-0.569175298	-1.372531808	-0.189589155	-0.431977243
8	-0.513901068	-0.392543811	-0.468470235	0.512078226	2.210151868	-0.290665039	-0.325748346	-0.556263671	1.254955165	0.586749491	0.891866565
9	-0.796115458	-0.228921702	-0.314709772	-0.199381833	-0.174254749	-0.388074253	-0.298884723	0.162152396	0.254167726	0.142967012	-0.69299846
10	-1.010580198	0.514390034	0.44846563	-0.614322649	-0.717625086	-0.461399429	-0.283522065	-0.580350031	0.400761067	-0.681577886	-0.343917911
11	-0.143725852	-0.572421291	-0.560125484	-0.618716223	-0.700038562	0.757844853	-0.46224822	-0.586890935	0.024050925	-0.884041746	3.057593454
12	0.345368729	3.543215113	2.626042016	-0.608867478	-0.554209487	-0.412841326	2.956905831	1.339916898	-0.10489716	0.022889289	-0.871869691
13	-0.230618006	-0.454543766	-0.469482594	0.830236944	1.577285488	-0.273329937	-0.385935323	-0.515768494	0.831757799	0.71777601	0.056556666
14	-0.593227446	-0.574800383	-0.552479604	0.198232335	-0.118069197	-0.513579886	-0.127055051	2.852305895	-1.255620319	-0.743172368	-0.371991023
15	-0.571647465	0.183606983	0.342158963	1.04406798	0.010692678	-0.489867015	-0.368705524	-0.185765498	0.367618443	0.97676371	0.827717814
16	-0.784660338	-0.310373122	-0.401074704	1.661182985	2.735160778	0.127754476	-0.587390462	-0.54877327	1.370768375	0.150210199	-0.046315442
17	-0.106493569	-0.579219592	-0.566062471	-0.563957071	-0.26108176	-0.605358037	-0.436849751	-0.570587044	-0.327947707	-0.316299405	-0.994961059
18	1.486158257	-0.536903964	-0.495700773	-0.614424713	-0.721910247	2.996565966	-0.597325258	-0.605207812	0.444344224	-0.26235465	-0.361170938
19	-0.02922192	-0.309748093	-0.279134166	-0.60067132	-0.711692055	2.89259423	-0.49736414	-0.599184042	-0.369554574	0.041578785	-1.36584175
20	1.749603719	-0.127782769	-0.140998234	-0.488357888	-0.455596432	-0.493930149	-0.467871748	-0.499758587	1.34994347	0.008073467	-0.120918361
21	2.833365135	-0.386315288	-0.356165311	-0.615183394	-0.694755865	-0.399709406	-0.470314422	-0.605507952	-0.131106634	-0.475993158	0.798279764
22	-0.798165912	-0.460185011	-0.486989066	-0.067190501	1.051509265	-0.09647391	-0.186276565	-0.476100659	0.757682005	-0.09852665	-0.015195871
23	-0.084116918	2.475753658	3.428397816	3.579299435	0.794469289	-0.530228744	-0.34783661	-0.579422543	1.155912119	3.753578293	1.727761655
24	-0.102107423	0.485746644	0.431062113	0.485450795	1.455602961	1.52637046	1.01035713	-0.246542506	0.856447918	1.176275055	-0.888921581
25	-0.714697342	-0.489919289	-0.531218471	-0.94139573	-0.821291779	-1.511080968	-1.087637457	-0.292497858	-0.350930665	-0.064610107	-1.061243117
26	-0.842912202	-0.65171456	-0.602345727	-0.387207189	-0.793139061	-0.309121221	-1.482993558	0.22527219	-0.679631822	-0.775854094	0.073647246
27	-0.459891353	-0.157291948	-0.12811027	2.13814198	0.634584833	1.523837161	0.117464139	-0.218321171	-0.163290387	-0.115599475	-0.92090419
28	-0.079726149	0.143654916	0.167162977	0.073288335	0.075833503	0.049722412	1.422213032	-0.358788637	-0.15644737	-0.356501695	-0.394452362
29	-1.011758717	-0.544693747	-0.51670658	-0.544200981	-0.755897609	-0.392314477	-0.578968279	-0.22313502	-0.652319438	-0.817653163	0.139536308
30	-0.82234017	-0.28424761	-0.504725647	-0.872057285	-0.957079254	-1.862717651	-0.814760648	-0.247530902	-0.386946878	0.106825329	-2.065209939
31	2.937266696	0.024197368	0.178332455	0.576827293	-0.866661851	-1.073065988	-1.080080421	4.413514098	-1.616867419	-0.808974388	0.970330222
32	-0.207218611	-0.194798598	-0.187093757	0.013822317	0.016822736	-0.422512006	1.85033551	-0.192770446	0.172351239	0.077634679	0.009306258
33	-1.368156417	-0.562499645	-0.571502475	-1.010143232	-1.052712642	-0.872668588	0.329239637	-0.296615156	-0.765482261	-1.141526932	-0.484071802
34	1.580975261	-0.653313595	-0.549290008	-0.854647557	-0.477516368	-0.314658547	-0.269942383	-0.268006465	-0.221051975	-0.622141592	2.939519614
35	-0.329075402	0.240533376	0.25308969	-0.257122863	0.045698664	1.021031091	1.026524992	-0.342202088	-0.744487103	0.057052293	-1.019527349
36	0.283866819	0.025543504	0.093792234	-0.105491176	0.107653449	-0.311927103	-0.036343818	0.242936755	-0.006451716	0.145846027	0.175703572
37	0.133110455	-0.592910584	-0.571349986	-0.904971907	-0.698302747	-0.44789354	0.391907248	-0.266490988	-0.461132635	-0.949778144	0.18416456
38	0.425137081	-0.39655783	-0.458562763	0.58737744	-0.350972817	-0.572994353	1.479611233	-0.295852841	0.348691388	0.317832536	0.04345619
39	0.413908519	0.282057208	0.145298861	-0.41952548	-0.004351556	-0.425633239	-0.147495297	-0.045703273	-0.04119959	-0.121493948	1.078127642
40	-0.435969151	-0.336534034	-0.307427288	-0.327590836	0.040718446	0.053997186	0.703154634	-0.221361187	-0.365088365	-0.565949368	0.742874365
41	0.530375219	0.545284402	0.650656755	0.077906664	1.789759937	1.291542433	-0.652936619	-0.21091965	3.153348133	3.016252394	-0.05298129
42	1.644104139	4.165649946	4.157291174	3.119527361	2.9533924	0.752499539	-2.009586838	-0.414029537	1.759431287	1.727245417	0.278767886
43	-0.36478241	-0.345546571	-0.299095287	0.123232027	0.514876854	0.080764884	-0.045519253	-0.332026435	1.164735662	1.149724167	-0.203238627
44	-0.280731285	-0.594554288	-0.539181489	-0.819647289	-0.88123385	0.213503583	-0.510129832	-0.352565596	-0.399057994	-0.866415094	0.908544685
45	-0.929377556	-0.108085064	-0.310076511	0.248427312	0.024215749	2.003318933	0.385586848	-0.056363285	-0.44462001	-0.568189899	-0.453428291
46	-1.161725571	-0.5947186	-0.553789404	-0.746870439	-0.987182915	-0.158281091	-0.130766693	-0.406874312	-0.730137422	-0.860858595	-1.491382247

Additional File 5 : Normalized expression values of microRNAs associated with hepatocellular carcinoma (page 3)

sl No.	hsa-miR-143-5p	hsa-miR-143-3p	hsa-miR-145-5p	hsa-miR-125a-5p	hsa-miR-136-3p	hsa-miR-146a-5p	hsa-miR-185-5p	hsa-miR-186-5p	hsa-miR-200a-3p	hsa-miR-151a-5p	hsa-miR-151a-3p
1	-1.133674165	-0.967492954	-0.622658921	-0.547246512	-0.953867472	-0.386581071	2.962179934	2.402079201	-0.797569292	-0.057026083	2.849912437
2	-0.953849556	-0.741006377	-0.580562747	-0.722936531	-0.819552127	-0.664569832	0.035032469	0.656222454	-0.76041177	-0.600073781	-0.002218806
3	1.044135145	0.704853897	-0.026936248	-0.204977469	0.547689889	-0.318891097	-0.747417994	-0.261123281	-0.519457415	-1.6916885	-1.06707782
4	-0.242577948	-0.510667284	-0.389463137	-0.339069342	-0.527868841	-0.544964181	-0.431985348	-0.306579133	-0.66253392	-0.005771116	0.548622051
5	-1.245636916	-0.975693985	-0.707465902	-0.817961474	-0.791968044	-0.773283252	-0.023175818	-0.639447153	-0.58195728	-1.598325121	-0.735366898
6	1.396160635	0.423106886	-0.205417762	0.020695395	-0.135371335	0.618641555	0.222164272	0.159372334	-0.263822605	-0.189898705	-0.069705774
7	-0.828288611	-0.577195603	-0.570857013	0.92944723	-0.488844554	2.825354958	1.553757838	3.173472698	1.100367235	0.312618477	1.050471377
8	1.179765003	3.213755337	2.585563336	1.180901014	1.790541326	0.510275161	-0.038806726	0.01044123	1.192758199	0.059452174	-0.423282427
9	0.951718391	0.236955486	-0.040564585	-0.17734672	0.161997521	-0.662548276	0.078146391	-0.57389946	-0.745965805	-1.103659118	-0.942602052
10	-0.944644206	-0.958425529	-0.67996588	-0.715355977	-0.952292918	2.715274975	-1.05871103	-0.555629775	-0.745161648	-0.323959544	-0.854544581
11	-0.839702057	-0.743859164	-0.426784527	-0.654670774	-0.877667795	-0.778424276	-1.081276707	-0.829786988	-0.641877203	0.444554299	0.18983786
12	-0.174846047	-0.103219606	-0.491521791	-0.708460616	-0.625054318	-0.62168999	-0.372411039	1.061090604	-0.430947024	-0.380424797	-0.149526933
13	0.593772436	1.30662726	1.139924	0.749607798	2.284290399	0.608215418	-0.110213871	0.045488303	2.349654963	-0.193702636	-0.574421283
14	0.299341809	0.25705027	-0.345993996	-0.130145216	0.015447477	-0.478266973	-0.840352574	-0.708925335	-0.100352265	-0.580842441	-1.271976886
15	1.437730268	0.662658575	-0.095789012	-0.15692355	1.584264411	-0.038001047	0.041636037	-0.297649121	2.093167187	-0.455542569	-0.13950858
16	1.407157122	1.1776673	2.96455487	3.571655334	1.942344089	0.284146447	-0.505404523	-0.63764495	-0.016789969	1.446367499	-0.458268567
17	-0.322847699	-0.234445782	-0.404154264	-0.422525326	-0.009195856	-0.725493498	-1.065472059	-0.720863182	-0.613110711	-0.900505882	-1.229304899
18	-1.370716087	-1.046080458	-0.704133323	-0.756253141	-0.971778841	-0.338908523	-0.598248972	-0.516682954	-0.587655846	0.10372453	-0.330994638
19	-0.95890594	-0.81981367	-0.593187804	-0.430504034	-0.72764149	-0.121812411	0.37047614	-0.662363587	-0.776638222	-0.60392653	1.440833637
20	-0.002198214	-0.181520784	-0.388862186	-0.454266473	-0.087191804	-0.756603852	-0.413211609	0.217644465	-0.624420009	1.219215437	0.172332799
21	-0.838006837	-0.818033355	-0.630788448	-0.796528397	-0.756948776	-0.678358992	2.096654119	-0.490822373	1.883777605	1.307595901	0.573111437
22	-0.018744624	0.171343157	0.895770694	1.130396035	0.300473113	0.692202397	-0.485758553	-0.381371994	0.36856747	1.603119892	-0.206647735
23	1.564858099	0.523436383	0.319294645	0.452468747	0.098195948	-0.365713639	0.412399624	-0.143022002	-0.119621675	2.188698613	1.630326281
24	0.818797831	1.18613462	1.145312321	1.788434365	0.363229379	0.283895218	1.193088695	1.535632153	1.047957128	-0.078799594	2.258850445
25	-1.028726274	-1.057432983	-1.240014357	-1.30600781	-1.069516954	1.299076506	0.228223236	-0.540580611	-0.81958947	-1.213110646	-1.089185523
26	-1.022558471	-0.85453444	-0.861087544	-0.945645918	-0.541128009	-0.540790975	-1.684142421	-0.694717351	-0.836110831	-0.798149624	-0.609149657
27	1.423174395	1.733788245	1.892962511	1.245745439	0.305887731	-0.577258502	-0.293150284	0.655811737	-0.624346004	-0.958773963	1.220101948
28	-0.082714302	0.108302273	0.30941723	0.783222758	-0.743534473	-0.000931815	1.463042254	0.509420799	0.15738173	2.56526163	1.405155588
29	-0.763223036	-0.946379477	-0.780973094	-0.755502233	-0.293950124	-0.83290092	-1.201658684	-0.808496784	-0.731742552	-0.509844246	-1.000644644
30	-1.495238102	-1.320993433	-1.322466168	-1.444232816	-1.204604328	-0.570175791	1.33227164	-1.659577631	-0.950306064	-1.463336583	-1.642741336
31	0.739575192	-0.664268315	-0.800692712	-0.904430244	-0.856623778	-0.991228705	0.475388948	0.745961115	-0.641607789	0.995431423	0.727021751
32	0.96025615	1.060980899	1.144002528	0.228461738	-0.334324313	-0.631415129	-0.304952903	-0.99869417	-0.36523404	-0.032265065	-0.456083017
33	-1.248106303	-1.29262194	-0.979597951	-0.990941877	-0.48034676	-0.880382749	-1.614466778	-1.712570737	-0.954036392	-0.287213857	-0.890679166
34	-0.603137962	-0.320588143	-0.6241257	-0.281358734	2.478510022	-0.949906934	1.390614602	-0.107469091	1.346481978	-1.704279515	-0.524727022
35	0.328433752	1.416318582	0.111928688	-0.521484511	1.44906621	-0.418739709	-0.263007272	0.303800236	-0.096016888	-0.071117446	-0.042383265
36	0.037599714	0.491783269	0.130401774	-0.078691525	0.53380074	0.108655682	0.233858415	0.511487317	1.786640583	0.940840474	0.27422608
37	-1.015669135	-0.962360057	-0.918634909	-0.803499817	1.563107858	-0.598717496	-1.456544255	-0.9868198	-0.188720603	-0.516190522	-0.939131894
38	-0.393038417	-0.398557235	-0.633444463	-0.226939297	1.427924093	0.435058191	0.455946816	0.308927985	-0.317890352	-0.130507693	-0.304740733
39	-0.290497226	0.298256545	-0.034361937	0.130988603	0.399340939	0.014840669	0.293238619	0.461522955	1.872091704	1.206977956	-0.006580783
40	0.106135656	-0.644556906	-0.250455318	0.239206064	-0.862748824	-0.389628473	0.285441655	0.363242887	-0.391693889	-0.174703155	-0.58892125
41	1.162362282	0.923853013	1.601520833	0.959950407	-0.941208972	3.236648104	0.372608509	1.63714096	0.578152405	0.557302792	1.601243415
42	2.145666828	1.393746424	0.708427686	1.858993519	0.498146027	1.007446326	1.050061539	1.463299979	2.164973194	0.478099635	0.670790678
43	1.175357291	0.593087395	0.945482995	1.233101195	-0.243699701	-0.000880966	0.05278864	0.857283706	-0.682295512	-0.143392683	-0.053011902
44	-1.110321265	-1.233365708	-0.92697768	-0.964604274	-0.632673327	-0.410611518	-1.50488742	-1.272591536	-0.747106244	0.002014817	-0.696256627
45	0.155871403	0.489407371	1.383375267	0.755234965	-0.814653438	1.407948986	-0.503763549	-0.572014116	-0.606982092	1.335755864	0.686846913
46	-1.008862331	-1.090120496	-0.903683145	-0.931669033	-0.351136134	-0.829392896	-1.369102701	-1.071472295	-1.095797786	0.343984043	-0.416475501

Normalized expression values of microRNAs associated with hepatocellular carcinoma (page 4)

sl No.	hsa-miR-92b-3p	hsa-miR-151b	hsa-miR-21-5p	hsa-miR-17-5p	hsa-miR-122-5p	hsa-miR-101-3p	hsa-miR-125b-5p	Class
1	-0.415064534	-0.053978448	-1.065814533	-0.124393289	1.733612148	-1.570616745	-0.188157921	HT
2	-0.402885694	-0.571753222	-0.233831541	0.167922931	0.977485079	-0.645824161	-0.558534559	HT
3	-0.269738704	-1.701885249	0.648144254	-0.766105292	-0.565268062	0.450813853	-0.161703018	HT
4	-0.430105326	-0.030131763	-0.601101824	-0.683271572	0.773178159	-0.223598342	0.418289181	HT
5	-0.48146726	-1.588307147	-0.056865871	-0.314300415	1.365789403	0.593236632	-1.024593354	HT
6	-0.037841536	-0.162026119	2.522646626	0.55302873	-1.711325436	0.876751681	-0.878921169	HT
7	0.603819014	0.308330649	0.392814915	3.623707499	0.397736233	1.520852515	-1.175123308	HT
8	-0.008073999	0.076381467	-0.445761613	-0.370101337	-1.51369708	2.657590482	1.478175956	HT
9	-0.306011498	-1.138446304	-0.412513273	-0.661630558	0.398497173	0.337005274	0.027076695	HT
10	-0.455117542	-0.37759946	-0.3758315	-0.288110844	0.681073535	-0.254052094	-0.748917039	HT
11	-0.451826251	0.347308674	-1.650371577	-0.424534319	0.887752616	-1.155119775	0.578701358	HT
12	-0.439969068	-0.221402384	-0.307563774	-0.306284141	-0.570876334	0.505292603	-0.039177116	HT
13	0.033207738	-0.149313127	-0.635316215	-0.239945208	-0.239420363	0.794200316	1.254442286	HT
14	-0.272137734	-0.59505483	0.343682313	0.151031257	-0.210273196	-0.090321631	-0.48598285	HT
15	-0.196883831	-0.502309185	0.769071591	1.4920227	-1.025146052	0.140943001	-0.511094345	HT
16	0.509132026	1.435081622	-0.243132406	-0.541125034	-0.489821269	-1.119690276	3.034056753	HT
17	-0.362273437	-0.862576724	-1.217574695	-0.41759615	0.874733205	0.270245661	-0.197407598	HT
18	4.363065025	0.100013741	-0.689355797	-0.719871162	0.586783719	-0.65166214	-0.113754702	HT
19	-0.420964371	-0.609426194	1.121790511	-0.63091788	0.161158487	-1.845576423	-0.935082094	HT
20	-0.37326273	1.110114153	0.336268971	-0.471685292	-0.33284344	-0.40772921	-0.168179061	HT
21	-0.440916548	1.210320699	1.509316013	-0.457704752	-1.253449144	0.490853937	-0.960598783	HT
22	0.011469807	1.768139997	-1.14977324	-0.088393865	0.860684594	-0.60912722	1.41695696	HT
23	0.243846455	2.208519155	1.441072664	1.518257994	-1.786363976	-0.06446794	-0.060472274	HT
24	1.473312522	-0.112793363	0.549200638	-0.689430379	-1.074274658	-0.34750587	1.148721432	HT
25	-1.00900627	-1.206170745	-0.598917762	0.257176168	1.233637636	-0.390241189	-1.601576164	HNT
26	-0.733550726	-0.859818094	-0.708374283	-0.557072147	0.898139036	0.365000356	-0.965769195	HNT
27	1.103011294	-0.992489584	-0.005808379	-0.201406609	-0.449961402	-0.754228796	1.602277415	HNT
28	0.927509654	2.475148945	0.053893614	-0.512625607	0.140544005	-0.361718682	0.75206205	HNT
29	-0.903484556	-0.47904458	-0.877147144	-0.630783252	0.590105278	0.549719175	-0.843563854	HNT
30	-1.138538781	-1.433394573	-1.159685584	-0.665463474	2.062067659	-1.126804854	-1.825233799	HNT
31	-0.670806746	0.829552201	1.673057719	2.96722713	-0.484387675	-0.113712309	-0.669926872	HNT
32	0.216366722	-0.048766649	-0.067852838	-0.646658414	-0.398392127	0.243503211	0.735558044	HNT
33	-1.021994701	-0.162655074	-0.993343157	-1.38754268	0.803723336	-0.120038492	-0.925534195	HNT
34	-0.787571287	-1.717792402	-0.455640223	1.803493433	-0.781524286	2.942713082	0.152861383	HNT
35	0.149950349	-0.008948038	0.214683216	-0.93594774	-0.120868431	-0.400518614	-0.241391157	HNT
36	-0.003414788	1.032966937	0.177508827	0.193383107	-0.145724798	0.166331343	0.1379975	HNT
37	-1.000139542	-0.501186211	-0.626202891	0.822955821	0.212857727	1.578411698	-0.585206637	HNT
38	-0.346949727	-0.151500448	0.61537014	0.143344294	-0.563605524	1.133878182	-0.26882067	HNT
39	0.015122582	1.299071564	0.501798539	0.223665682	-0.539211001	1.204807328	0.356769639	HNT
40	-0.489784456	-0.261178159	0.151788672	1.439344731	0.33266673	-0.280072474	0.299673703	HNT
41	1.804176151	0.651303344	-0.374960716	0.150312105	-0.643324834	-1.133393156	0.655538338	HNT
42	2.086656024	0.47931532	3.324629702	-0.616855269	-2.774792454	-0.507830649	1.404588132	HNT
43	0.596599712	-0.255720801	0.240046261	-0.436320494	-0.241733899	-1.166691136	0.591330882	HNT
44	-1.010660154	0.041440955	-0.897458664	-0.266913705	1.274581232	-0.541325759	-1.31006995	HNT
45	0.743196725	1.382659457	-0.736585688	-0.453882701	0.66947845	-0.940282397	1.399713973	HNT
46	-0.910644981	0.37556983	-1.158007584	-1.727775682	1.231372494	-0.917937894	-0.614029589	HNT
