# Supplementary material for: An approach to forecast human cancer by profiling microRNA expressions from NGS data
Source: BMC Cancer. 2017 Jan 25;17:77. doi: 10.1186/s12885-016-3042-2 (PMC5267436; doi:10.1186/s12885-016-3042-2)
Supplement: Additional file 6 — Normalized expression values of specific set of microRNAs associated with the carcinomas of the bladder. (PDF 43 kb) [file 12885_2016_3042_MOESM6_ESM.pdf]

**Additional File 6 : Normalized expression values of microRNAs associated with carcinomas of the bladder (page 1)**

| sl No. | hsa-miR-16-5p | hsa-miR-17-5p | hsa-miR-18a-5p | hsa-miR-19a-3p | hsa-miR-23a-3p | hsa-miR-26a-5p | hsa-miR-26b-5p | hsa-miR-96-5p | hsa-miR-99a-5p | hsa-miR-103a-3p | hsa-miR-192-5p | hsa-miR-10b-5p | hsa-miR-34a-5p |
|---|---|---|---|---|---|---|---|---|---|---|---|---|---|
| 1 | 0.010226116 | 0.008777757 | 0.001036628 | 0.00038107 | 0.002153558 | 0.006816924 | 0.005099918 | 0.000801562 | 5.11014E-05 | 0.076002354 | 0.004790389 | 0.005451787 | 0.000411731 |
| 2 | 0.005993093 | 0.001424266 | 6.87212E-05 | 3.09623E-05 | 0.002905925 | 0.009602089 | 0.00456052 | 0.000021145 | 0.001526215 | 0.024886138 | 0.001437104 | 0.008636216 | 0.000188039 |
| 3 | 0.014635878 | 0.011141044 | 0.001171678 | 0.000561309 | 0.012371884 | 0.010930373 | 0.007044499 | 0.000419899 | 0.000204899 | 0.088512214 | 0.000782081 | 0.005540942 | 0.000681074 |
| 4 | 0.010630001 | 0.003376491 | 0.000338838 | 0.000174769 | 0.004094589 | 0.018812046 | 0.01123872 | 0.000208058 | 0.002060373 | 0.051390425 | 0.001244784 | 0.006266719 | 0.000438706 |
| 5 | 0.005823054 | 0.001962721 | 0.000184471 | 0.000046796 | 0.002124133 | 0.006497188 | 0.002784703 | 1.35641E-05 | 0.001104793 | 0.022548905 | 0.00214787 | 0.006491763 | 9.15575E-05 |
| 6 | 0.020793007 | 0.001896394 | 5.04957E-05 | 0.000218815 | 0.007877329 | 0.015659277 | 0.014638142 | 0.001099684 | 0.000106602 | 0.088833157 | 0.004539002 | 0.025730364 | 0.001615862 |
| 7 | 0.03500873 | 0.002087606 | 2.13021E-05 | 1.61896E-05 | 0.059402195 | 0.118262464 | 0.035181703 | 0.000275223 | 0.007528164 | 0.107326816 | 0.02978375 | 0.002424179 | 0.001276422 |
| 8 | 0.013337863 | 0.010685533 | 0.001779108 | 0.000655461 | 0.002922353 | 0.011384546 | 0.00830105 | 0.000128479 | 4.35522E-05 | 0.138517788 | 0.002238583 | 0.005487578 | 0.000213406 |
| 9 | 0.00666603 | 0.005680051 | 0.000369996 | 0.000306975 | 0.002959969 | 0.010943348 | 0.008601394 | 0.00066274 | 3.45601E-05 | 0.057530337 | 0.003945948 | 0.010264344 | 0.000679004 |
| 10 | 0.00666603 | 0.005680051 | 0.000369996 | 0.000306975 | 0.002959969 | 0.010943348 | 0.008601394 | 0.00066274 | 3.45601E-05 | 0.057530337 | 0.003945948 | 0.010264344 | 0.000679004 |
| 11 | 0.76625094 | 0.505959876 | 0.462556042 | 0.489583436 | 0.70153526 | 0.645503244 | 0.592855631 | 0.505714025 | 0.435663037 | 0.64135967 | 0.492699778 | 0.864119739 | 0.573575991 |
| 12 | -0.296670622 | -3.066626296 | -1.881914496 | -1.513164497 | 0.561760869 | 0.502658587 | 0.325380529 | -0.61279294 | 0.447205687 | 0.026691253 | 0.403956377 | -1.523818198 | -0.73261022 |
| 13 | 0.755381094 | 0.502249082 | 0.409888184 | 0.473955291 | 0.684541446 | 0.636863277 | 0.571695622 | 0.504420381 | 0.437260798 | 0.611792849 | 0.490776655 | 0.785266005 | 0.519933068 |
| 14 | 0.772531187 | 0.477285551 | 0.309544474 | 0.459237685 | 0.70283114 | 0.646367932 | 0.566302756 | 0.501458473 | 0.440656856 | 0.612075766 | 0.491749088 | 0.8211119 | 0.56117812 |
| 15 | 0.70360765 | 0.364983446 | 0.365596676 | 0.495866734 | 0.692823324 | 0.63044713 | 0.571613705 | 0.503702423 | 0.434218258 | 0.575971489 | 0.482730206 | 0.711264218 | 0.579992364 |
| 16 | 0.817343692 | 0.672558299 | 0.544184075 | 0.538715803 | 0.728106859 | 0.668114241 | 0.607516985 | 0.498358956 | 0.445564285 | 0.676303013 | 0.49787496 | 0.848056123 | 0.561318714 |
| 17 | 0.211990675 | 0.542319646 | 0.542435731 | 0.543188526 | -0.990867787 | -0.63244192 | 0.096549274 | 0.495309895 | -0.451624647 | 0.547097058 | 0.436001683 | 0.883420842 | 0.51306913 |
| 18 | 0.784044802 | 0.57478485 | 0.496802962 | 0.502291586 | 0.711460439 | 0.6211014 | 0.563039141 | 0.500056601 | 0.438625583 | 0.671943959 | 0.493533227 | 0.696682823 | 0.527196939 |
| 19 | 0.607463289 | 0.243775304 | 0.213227104 | 0.352874296 | 0.675765173 | 0.608970476 | 0.542232549 | 0.480272111 | 0.42975154 | 0.477861432 | 0.472639005 | 0.064674012 | 0.322751285 |
| 20 | 0.330208523 | -0.169718514 | 0.055083961 | -0.30301613 | 0.502375665 | 0.535501508 | 0.446344236 | 0.456753265 | 0.411017265 | 0.408850858 | 0.471723646 | -1.531403866 | 0.241936594 |

**Additional File 6 : Normalized expression values of microRNAs associated with carcinomas of the bladder (page 2)**

| sl No. | hsa-miR-182-5p | hsa-miR-183-5p | hsa-miR-205-5p | hsa-miR-210-3p | hsa-miR-214-3p | hsa-miR-221-5p | hsa-miR-221-3p | hsa-miR-222-3p | hsa-miR-223-3p | hsa-miR-200b-3p | hsa-miR-23b-3p |
|---|---|---|---|---|---|---|---|---|---|---|---|
| 1 | 0.117263068 | 0.07143827 | 0.010329779 | 0.000436552 | 8.76024E-06 | 0.00074024 | 0.000852663 | 0.000845363 | 7.73821E-05 | 0.06144284 | 0.003359551 |
| 2 | 0.002865901 | 0.003188362 | 0.007164374 | 0.000169915 | 7.17419E-05 | 0.000809551 | 0.000512766 | 0.001399345 | 0.000135932 | 0.009779556 | 0.002731479 |
| 3 | 0.046300051 | 0.014943227 | 0.024351284 | 0.003591511 | 9.52349E-05 | 0.018068664 | 0.005988257 | 0.006338895 | 0.000264061 | 0.021901149 | 0.00587715 |
| 4 | 0.01334546 | 0.009971347 | 0.006920617 | 0.000236592 | 0.000340027 | 0.002826028 | 0.000779922 | 0.001811891 | 0.000859578 | 0.01310649 | 0.003078075 |
| 5 | 0.002409657 | 0.002191953 | 0.001145485 | 0.000073246 | 3.93358E-05 | 0.00036623 | 0.000244153 | 0.000991533 | 0.000334354 | 0.007139447 | 0.002752827 |
| 6 | 0.17727918 | 0.062738101 | 0.026785163 | 0.001088463 | 9.53808E-05 | 0.012062862 | 0.001026746 | 0.001660747 | 0.000824763 | 0.024288431 | 0.006143643 |
| 7 | 0.00842626 | 0.002966105 | 0.04311631 | 0.01589563 | 0.000284596 | 0.003617949 | 0.002239277 | 0.011403442 | 0.001210812 | 0.039022898 | 0.026756295 |
| 8 | 0.037001953 | 0.014820815 | 0.022159362 | 0.001726845 | 0.000119769 | 0.007475736 | 0.000801361 | 0.003072608 | 0.001432868 | 0.01149996 | 0.001215107 |
| 9 | 0.07635135 | 0.056030023 | 0.01240097 | 0.00113845 | 4.06589E-06 | 0.001002242 | 0.000587521 | 0.001091692 | 4.06589E-05 | 0.062919676 | 0.00241514 |
| 10 | 0.07635135 | 0.056030023 | 0.01240097 | 0.00113845 | 4.06589E-06 | 0.001002242 | 0.000587521 | 0.001091692 | 4.06589E-05 | 0.062919676 | 0.00241514 |
| 11 | 0.437944972 | 0.44804522 | 0.361467954 | 0.759613303 | 0.492947956 | 0.776031273 | 0.605247428 | 0.71193217 | 0.442381474 | 0.411105566 | 0.608279448 |
| 12 | -0.745505139 | -2.304490632 | -3.783732043 | -1.727201294 | 0.49014917 | 0.552207423 | 0.523170042 | 0.68741934 | 0.402971001 | -3.774042639 | 0.499805779 |
| 13 | 0.366370791 | 0.390269066 | 0.305083554 | 0.748988028 | 0.487053179 | 0.176046018 | 0.529445054 | 0.702350706 | 0.406263929 | 0.34190586 | 0.593954327 |
| 14 | 0.331835137 | 0.379660036 | 0.309414517 | 0.756755731 | 0.487905303 | 0.364762629 | 0.527685225 | 0.687955001 | 0.421660757 | 0.340972343 | 0.607723857 |
| 15 | 0.321712707 | 0.373396727 | 0.347043217 | 0.723455149 | 0.491336308 | 0.691880351 | 0.593413737 | 0.697932547 | 0.376108881 | 0.334046199 | 0.544450474 |
| 16 | 0.490968013 | 0.420808665 | 0.378230556 | 0.777122128 | 0.475190099 | 0.741420229 | 0.624223416 | 0.739130108 | 0.429791293 | 0.372710584 | 0.68776808 |
| 17 | 0.327445738 | 0.377690177 | -0.096453597 | -0.548105045 | 0.370455088 | -1.880369023 | -0.369316548 | -1.022585422 | 0.051158777 | 0.235176007 | -2.855819136 |
| 18 | 0.352198401 | 0.384834027 | 0.297067933 | 0.744079493 | 0.474996904 | 0.642652158 | 0.609175501 | 0.717885294 | 0.435089833 | 0.307405147 | 0.657540772 |
| 19 | 0.3568671 | 0.266212683 | -0.076125533 | 0.599269529 | 0.485490943 | 0.608045981 | 0.521432038 | 0.641596506 | 0.383077934 | 0.024459037 | 0.560210451 |
| 20 | 0.375503962 | 0.343230327 | -0.337538669 | 0.536737382 | 0.47985365 | 0.500749652 | 0.512121863 | 0.646233737 | 0.24337145 | -0.443077073 | 0.428032047 |

**Additional File 6 : Normalized expression values of microRNAs associated with carcinomas of the bladder (page 3)**

| sl No. | hsa-miR-125b-5p | hsa-miR-133a-3p | hsa-miR-141-3p | hsa-miR-143-3p | hsa-miR-144-5p | hsa-miR-145-5p | hsa-miR-126-5p | hsa-miR-126-3p | hsa-miR-185-5p | hsa-miR-195-5p | hsa-miR-200c-3p |
|---|---|---|---|---|---|---|---|---|---|---|---|
| 1 | 0.000414651 | 7.3002E-06 | 0.020425952 | 0.179246123 | 0.004949534 | 0.000470133 | 0.002449946 | 0.007906114 | 0.000849743 | 0.000420491 | 0.35121687 |
| 2 | 0.00979768 | 2.94519E-05 | 0.001767872 | 0.766048855 | 0.00430527 | 0.002261003 | 0.003408119 | 0.01855548 | 0.000220512 | 0.001727092 | 0.075089621 |
| 3 | 0.002248122 | 5.77181E-06 | 0.016595409 | 0.458019431 | 0.002320269 | 0.001004296 | 0.001243826 | 0.00900403 | 0.001034598 | 0.000603155 | 0.145484349 |
| 4 | 0.00886091 | 3.68561E-05 | 0.003823519 | 0.635517352 | 0.015956296 | 0.002685737 | 0.005730523 | 0.03338089 | 0.000844123 | 0.00260608 | 0.046395834 |
| 5 | 0.006387997 | 5.49345E-05 | 0.0009739 | 0.84394812 | 0.006684372 | 0.004203504 | 0.002713492 | 0.016527138 | 0.000238049 | 0.001438469 | 0.021170796 |
| 6 | 0.000572285 | 3.26042E-05 | 0.022246161 | 0.226950116 | 0.004808313 | 0.000392744 | 0.000471293 | 0.005504031 | 0.001223118 | 0.003832063 | 0.208519186 |
| 7 | 0.003787514 | 4.26042E-05 | 0.002883453 | 0.089292455 | 0.000572601 | 0.01243702 | 0.00221201 | 0.000510398 | 0.037311061 | 0.002483825 | 0.071001616 |
| 8 | 0.003691049 | 2.17761E-06 | 0.015622176 | 0.1497695 | 0.007689142 | 0.000111058 | 0.001580945 | 0.029700426 | 0.002867913 | 0.00060102 | 0.248071182 |
| 9 | 0.000412688 | 2.03295E-06 | 0.028530363 | 0.147878519 | 0.002535084 | 0.000319173 | 0.001211636 | 0.006135431 | 0.000748124 | 0.000333403 | 0.422862916 |
| 10 | 0.000412688 | 2.03295E-06 | 0.028530363 | 0.147878519 | 0.002535084 | 0.000319173 | 0.001211636 | 0.006135431 | 0.000748124 | 0.000333403 | 0.422862916 |
| 11 | 0.339624876 | 0.350330391 | 0.390738527 | -0.830898058 | 0.410272532 | 0.505741669 | 0.579888644 | 0.221316736 | 0.572376412 | 0.422540039 | 0.689535994 |
| 12 | 0.405979148 | 0.51502716 | -3.769505891 | 1.794336829 | -0.684669176 | 0.573087054 | -0.322856545 | -0.508407115 | 0.528806154 | 0.486351367 | -3.739285732 |
| 13 | 0.332742773 | 0.311798425 | 0.354802522 | -0.501874513 | 0.495246201 | 0.502847851 | 0.582859135 | -0.313378773 | 0.569538727 | 0.435810646 | 0.454570232 |
| 14 | 0.372152787 | 0.347415249 | 0.353644561 | -0.34255323 | 0.343810444 | 0.4793593 | 0.351603899 | 0.130982287 | 0.570878173 | 0.408344545 | 0.375155472 |
| 15 | 0.337325833 | 0.291339254 | 0.374933198 | -0.132301739 | -0.824724572 | 0.356807745 | 0.515745694 | 0.298857633 | 0.570289215 | 0.393332867 | 0.354960662 |
| 16 | 0.393449165 | 0.491849144 | 0.387085891 | -0.816262947 | 0.717296532 | 0.561243911 | 0.823566929 | 0.689307967 | 0.572709879 | 0.409440172 | 0.538504496 |
| 17 | 0.094307856 | -3.384073647 | 0.36957911 | 0.171158513 | 0.747480627 | -2.816973721 | 0.670013612 | 0.832859618 | 0.168701421 | 0.082885717 | 0.375018108 |
| 18 | 0.381318577 | 0.451350718 | 0.343942135 | -0.831181754 | -0.156053291 | 0.555863012 | 0.648687374 | 0.113028113 | 0.572906658 | 0.431721354 | 0.521778247 |
| 19 | 0.296916591 | 0.393506957 | -0.079066897 | -0.611115045 | -0.849333868 | 0.509135706 | -0.296590776 | -0.394409534 | 0.564534289 | 0.394527805 | 0.407607784 |
| 20 | 0.267470166 | 0.478904688 | -0.387122364 | -0.307689598 | -3.138063288 | 0.514433672 | -1.556852137 | -2.252851509 | 0.555981639 | 0.33467973 | 0.069215764 |

**Additional File 6 : Normalized expression values of microRNAs associated with carcinomas of the bladder (page 4)**

| sl No. | hsa-miR-155-5p | hsa-miR-29c-3p | hsa-miR-200a-3p | hsa-miR-335-5p | hsa-miR-429 | hsa-miR-451a | hsa-miR-409-3p | hsa-miR-146b-5p | hsa-miR-493-5p | hsa-miR-320c | Class |
|---|---|---|---|---|---|---|---|---|---|---|---|
| 1 | 0.000213166 | 0.001249794 | 0.01814099 | 0.000182505 | 0.010494764 | 0.000883324 | 0.000106583 | 0.01148759 | 8.32222E-05 | 5.84016E-06 | BT |
| 2 | 0.000137442 | 0.001302682 | 0.002303293 | 4.15348E-05 | 0.000999101 | 0.001768627 | 0.000129135 | 0.01986949 | 0.000111011 | 1.73691E-05 | BT |
| 3 | 0.000471846 | 0.000137081 | 0.00551064 | 0.000336208 | 0.003093692 | 0.001030269 | 0.000186141 | 0.049727065 | 0.000206342 | 2.02013E-05 | BT |
| 4 | 0.00045654 | 0.001251917 | 0.004639108 | 0.000181902 | 0.002787983 | 0.006440299 | 0.000311493 | 0.064199689 | 0.000312682 | 2.97226E-05 | BT |
| 5 | 0.000737885 | 0.000678203 | 0.002105821 | 2.64499E-05 | 0.001649391 | 0.003310989 | 0.000140388 | 0.017429148 | 5.62909E-05 | 1.49205E-05 | BT |
| 6 | 0.000364691 | 0.002536006 | 0.007299434 | 0.000241257 | 0.002536006 | 0.001812235 | 5.04957E-05 | 0.01346552 | 3.36638E-05 | 8.97701E-05 | BT |
| 7 | 0.000469498 | 0.112049918 | 0.034449762 | 0.001544829 | 0.017214656 | 0.038251762 | 0.000199388 | 0.019581746 | 5.62376E-05 | 0.000113327 | BT |
| 8 | 0.003871791 | 0.000139367 | 0.004222386 | 7.62164E-05 | 0.002421503 | 0.001256481 | 4.13746E-05 | 0.23109671 | 6.09731E-05 | 8.49268E-05 | BT |
| 9 | 0.000284612 | 0.002433436 | 0.030069303 | 8.33508E-05 | 0.011740263 | 0.000652576 | 2.84612E-05 | 0.021069452 | 2.64283E-05 | 1.62636E-05 | BT |
| 10 | 0.000284612 | 0.002433436 | 0.030069303 | 8.33508E-05 | 0.011740263 | 0.000652576 | 2.84612E-05 | 0.021069452 | 2.64283E-05 | 1.62636E-05 | BT |
| 11 | 0.396271157 | 0.645523524 | 0.441448408 | 0.57154759 | 0.423863163 | 0.606978659 | 0.522550159 | 0.665785175 | 0.595047531 | 0.445787245 | BNT |
| 12 | 0.352338743 | 0.481513322 | -3.670067133 | 0.190742613 | -3.69324176 | 0.581667375 | 0.465693869 | 0.515642892 | 0.520611261 | 0.438821308 | BNT |
| 13 | 0.388744954 | 0.647352644 | 0.396638052 | 0.551599726 | 0.379803474 | 0.606154871 | 0.492579975 | 0.660483099 | 0.54884087 | 0.444685804 | BNT |
| 14 | 0.393928799 | 0.644163163 | 0.406199492 | 0.554572089 | 0.377289485 | 0.593861418 | 0.513243443 | 0.626379542 | 0.577838804 | 0.443880594 | BNT |
| 15 | 0.398077125 | 0.63597876 | 0.412990441 | 0.554458088 | 0.365130484 | 0.498980455 | 0.51848351 | 0.667009751 | 0.599201576 | 0.441443366 | BNT |
| 16 | 0.397786849 | 0.654518589 | 0.440374975 | 0.571197155 | 0.420576028 | 0.615352006 | 0.516734915 | 0.672310359 | 0.587065954 | 0.446305019 | BNT |
| 17 | 0.39326002 | -1.256196258 | -0.139406452 | 0.407617201 | 0.082850488 | -2.633492651 | 0.500603959 | 0.635585657 | 0.590372809 | 0.362085981 | BNT |
| 18 | 0.398858914 | 0.649076174 | 0.372983207 | 0.568202075 | 0.346847067 | 0.600341289 | 0.521211685 | 0.884542741 | 0.577385277 | 0.445300132 | BNT |
| 19 | 0.368142725 | 0.599645093 | -0.081609112 | 0.54356532 | -0.319614195 | 0.566829077 | 0.466740937 | 0.58202032 | 0.558882741 | 0.439283703 | BNT |
| 20 | 0.279624986 | 0.535458556 | -0.721001783 | 0.518829038 | -0.585093692 | 0.509126208 | 0.439956454 | 0.13103822 | 0.408340669 | 0.435755771 | BNT |
