# Supplementary material for: An approach to forecast human cancer by profiling microRNA expressions from NGS data
Source: BMC Cancer. 2017 Jan 25;17:77. doi: 10.1186/s12885-016-3042-2 (PMC5267436; doi:10.1186/s12885-016-3042-2)
Supplement: Additional file 7 — Normalized expression values of microRNAs associated with lung cancer and hepatocellular carcinoma (Test data sets). (PDF 48 kb) [file 12885_2016_3042_MOESM7_ESM.pdf]

Normalized Expression values of microRNAs associated with Lung Cancer (Independent Test Set) (page 1)

sl No.	hsa-let-7c-5p	hsa-let-7d-5p	hsa-let-7e-5p	hsa-let-7g-5p	hsa-let-7i-5p	hsa-miR-7-1-3p	hsa-miR-17-5p	hsa-miR-18a-5p	hsa-miR-18a-3p	hsa-miR-19a-3p
1	0.759859697	2.351264679	1.581500572	0.916132299	0.0604969	-0.00621338	-0.008099809	-0.279324526	0.086010899	-0.666874215
2	-1.39517178	-0.585827496	-1.102009541	-1.320450017	-0.508340078	-0.50675474	0.246948428	0.539796929	0.260903528	0.340999995
3	-1.195043495	-0.237214637	-1.029035699	0.359750572	-0.49166756	-0.505784196	-0.292543005	-0.097009735	-0.477347631	-0.410612853
4	0.265498823	-0.377361396	-1.189513314	-0.52740104	-0.245488429	-0.355148418	0.022798608	1.219243514	1.063455417	1.894250802
5	0.113820106	-1.146973632	0.642547813	-0.900611591	-0.765339339	-0.456625541	-0.715063807	-0.867995955	-0.927354248	-0.699427231
6	-1.174950013	-0.320466637	1.173793845	-0.875190902	-1.117074779	-0.781158673	-0.685064587	-0.87913424	-0.816669091	-0.736428199
7	0.448999357	0.457659546	0.070598361	1.676110516	2.280851927	2.499284569	2.489287818	1.842296195	2.062701684	-0.440104611
8	1.008523241	0.311583238	-0.118640892	0.781683495	0.606240381	0.436829232	-0.769657425	-0.98614966	-0.607362379	-0.689493467
9	1.168464063	-0.452663665	-0.029241144	-0.110023332	0.180320977	-0.324428854	-0.288606221	-0.491722521	-0.64433818	1.40768978

Normalized Expression values of microRNAs associated with hepatocellular carcinoma (Independent Test Set)

sl No.	hsa-let-7c-5p	hsa-let-7d-5p	hsa-let-7e-5p	hsa-miR-18a-5p	hsa-miR-23a-3p	hsa-miR-27a-3p	hsa-miR-29a-3p	hsa-miR-30a-5p	hsa-miR-100-5p	hsa-miR-16-2-3p
1	0.765563248	0.989763258	0.814972326	-0.520691944	1.189604363	-0.45600468	1.067940663	-0.819792608	-0.841704706	-0.571160496
2	-0.866275507	-0.87581318	-0.874877707	1.342869425	-1.063406044	0.710346591	-0.853870481	1.189878713	0.561121461	1.494154625
3	0.96096257	0.732590556	0.915563998	-0.953119044	0.40915397	-1.189037837	0.637107006	-0.834522408	-0.843911714	-0.549014383
4	-0.860250311	-0.846540634	-0.855658617	0.130941563	-0.53535229	0.934695926	-0.851177188	0.464436303	1.124494959	-0.373979746

Normalized Expression values of microRNAs associated with Lung Cancer (Independent Test Set) (page 2)

hsa-miR-19b-3p	hsa-miR-20b-5p	hsa-miR-24-1-5p	hsa-miR-29a-3p	hsa-miR-30c-2-3p	hsa-miR-33a-5p	hsa-miR-92b-3p	hsa-miR-1-3p	hsa-miR-23a-3p	hsa-miR-101-3p
-0.674689208	-0.784220607	-0.136834607	-0.50692254	0.06024948	-0.321903783	0.167210932	0.074631611	-0.554435118	0.813876552
0.15150369	1.156420594	-0.404699327	-0.728761326	-0.530290009	1.546279006	-0.830071488	-0.612055849	-0.324870049	-0.94052827
-0.339638311	0.237980058	-1.210931787	-0.183363262	-0.420688841	-0.521692103	-0.157572406	-0.51809953	-0.082960876	-0.352946527
1.29861296	-0.266372366	0.519831477	0.338334171	-0.347311027	0.507165821	-0.492540556	-0.685250765	1.106578957	-0.218505801
-0.636246559	-0.134285258	0.167792125	-0.082505206	-0.534194004	-0.823571232	-0.619464424	-0.35134628	-0.713591983	-0.851539894
-0.726931514	-1.12644396	-1.323428966	-0.193673716	-0.598964348	-1.187778626	0.663605466	-0.309269899	-0.911499992	-0.657525358
-0.420827575	1.941931837	1.436414953	-0.771913852	-0.298836577	1.574212404	2.351340985	-0.60195352	-0.380823512	-0.4298073
-0.686206495	-0.92285248	-0.468792207	-0.377431008	0.086100268	-0.62113811	-0.737176966	0.555536919	-0.321757553	0.4635969
2.034423012	-0.102157816	1.420648342	2.506236739	2.583935057	-0.151573376	-0.345331543	2.447807312	2.183360125	2.173379697

Normalized Expression values of microRNAs associated with hepatocellular carcinoma (Independent Test Set)

hsa-miR-197-3p	hsa-miR-148a-3p	hsa-miR-30d-5p	hsa-miR-34a-5p	hsa-miR-182-5p	hsa-miR-183-5p	hsa-miR-199b-3p	hsa-miR-204-5p	hsa-miR-210-3p	hsa-miR-217
-0.598076955	-0.80962468	-0.916669045	1.434343246	-0.764425762	-0.786703091	1.34525647	-0.648425348	1.087636961	0.722937785
-0.014657351	1.134469169	0.385305694	-0.671737906	0.184341338	0.209812	-0.761214472	-0.163417701	0.595542615	-0.860542003
-0.801416096	-0.871197812	-0.701293991	-0.6980393	-0.761961247	-0.753349901	0.177172474	-0.648425348	-0.690215926	0.998146222
1.414150402	0.546353324	1.232657342	-0.064566039	1.342045671	1.330240992	-0.761214472	1.460268397	-0.99296365	-0.860542003

Normalized Expression values of microRNAs associated with Lung Cancer (Independent Test Set) -page 3

hsa-miR-103a-3p	hsa-miR-197-3p	hsa-miR-135b-5p	hsa-miR-138-5p	hsa-miR-146a-5p	hsa-miR-148a-3p	hsa-miR-153-3p	hsa-miR-186-5p	hsa-miR-187-3p
-0.178235283	1.324156946	0.430874628	0.123422459	-0.442841572	0.119168797	-0.677194986	-0.588691335	2.069974741
-0.116267189	-0.699261026	-0.985220098	-0.526579928	-0.780967473	-0.897975499	0.5412177	-0.046889826	0.020286242
-0.496320582	-0.966490668	-0.822666765	-0.56727279	0.477406505	0.422497405	-0.95590128	-0.045507049	-0.788813427
0.865185501	-0.541404886	-0.757604851	1.952773471	-0.372702204	1.895833126	0.499285386	1.015224211	-0.617803249
-0.617084984	-0.690700955	0.167447113	0.779281739	-1.015717158	-0.443402555	-0.872381075	-0.606379436	-0.363715019
-1.621843289	-0.792178369	1.08647541	-1.083668451	-1.062442782	-1.406560865	-0.935000376	-0.827661566	-0.153643606
0.096538626	0.428320589	1.864544098	-0.986654004	1.860427495	-0.785383768	0.165965569	-0.651980878	-0.893487034
0.085072987	1.794999438	-0.952343435	-0.465976682	1.063345127	0.633029612	0.135363064	-0.466841845	-0.500187667
1.982954211	0.142558931	-0.031506099	0.774674188	0.273492063	0.462793748	2.098645999	2.218727722	1.22738902

Normalized Expression values of microRNAs associated with hepatocellular carcinoma (Independent Test Set)

hsa-let-7g-5p	hsa-let-7i-5p	hsa-miR-30b-5p	hsa-miR-125a-5p	hsa-miR-146a-5p	hsa-miR-185-5p	hsa-miR-186-5p	hsa-miR-200a-3p	hsa-miR-151a-5p
0.96423841	-0.810045874	-0.765908463	-0.832539253	-0.829203149	0.937794096	-0.818148888	-0.847278966	-0.808368876
-0.84996864	0.912162791	0.251440743	1.12934035	0.475870057	-0.864472058	0.407378185	-0.847278966	0.424556856
0.761787825	-0.918978614	-0.795814872	-0.851046733	-0.829203149	0.79115002	-0.814494329	1.100728579	-0.830990711
-0.876057595	0.816861697	1.310282591	0.554245636	1.182536241	-0.864472058	1.225265032	0.593829354	1.214802731

Normalized Expression values of microRNAs associated with Lung Cancer (Independent Test Set) -page 4

hsa-miR-194-5p	hsa-miR-199b-3p	hsa-miR-200a-3p	hsa-miR-210-3p	hsa-miR-215-5p	hsa-miR-21-5p	class
-0.250880274	0.457188752	0.221207458	0.007212323	1.996646101	-0.118231002	LT
-0.172747756	0.629713222	0.060063559	-0.162769372	-1.09825914	0.930060341	LT
0.287786313	-1.213905196	-0.795568949	-0.280139109	-1.125893436	0.012507687	LT
1.047700959	-0.770900876	-0.048284588	-0.742662681	-0.415152534	-1.28816684	LT
-0.582568165	-0.186887171	0.669440446	1.61083221	-0.732447913	0.821818069	LT
-0.920469442	-0.882069531	-0.076051226	-0.61526496	0.01461271	1.561446788	LT
2.09837591	-0.700441685	2.115426714	1.759191165	0.076663793	0.001479848	LT
-0.776682315	0.851083933	-1.27321026	-0.916312212	0.625145189	-0.45895035	LT
-0.73051523	1.816218552	-0.873023154	-0.660087365	0.65868523	-1.46196454	LT

Normalized Expression values of microRNAs associated with hepatocellular carcinoma (Independent Test Set)

hsa-miR-151a-3p	hsa-miR-92b-3p	hsa-miR-151b	hsa-miR-21-5p	hsa-miR-17-5p	hsa-miR-122-5p	hsa-miR-101-3	hsa-miR-125b-5	**class**
-0.850789156	0.481173723	-0.807980472	-0.669475905	1.47929043	-0.336684519	1.039810286	-0.651689439	HT
0.65187857	-0.840100616	0.423136784	1.369397413	-0.525600519	-0.576836174	0.329858498	-0.155341896	HT
-0.857474546	1.179035492	-0.830825557	-0.814507672	-0.275994645	1.490356867	-0.02453459	-0.651358728	HT
1.056385132	-0.820108598	1.215669245	0.114586164	-0.677695266	-0.576836174	-1.34513419	1.458390064	HT
